# Electrochemical Reduction of Nitric Oxide with 1.7% Solar‐to‐Ammonia Efficiency Over Nanostructured Core‐Shell Catalyst at Low Overpotentials

**DOI:** 10.1002/advs.202201410

**Published:** 2022-08-18

**Authors:** Sridhar Sethuram Markandaraj, Tamilselvan Muthusamy, Sangaraju Shanmugam

**Affiliations:** ^1^ Department of Energy Science & Engineering Daegu Gyeongbuk Institute of Science & Technology (DGIST) Daegu 42988 Republic of Korea

**Keywords:** ammonia, core‐shell nanostructure, Ni@NC, nitric oxide reduction, PV‐electrolyzer cell

## Abstract

Transition metals have been recognized as excellent and efficient catalysts for the electrochemical nitric oxide reduction reaction (NORR) to value‐added chemicals. In this work, a class of core–shell electrocatalysts that utilize nickel nanoparticles in the core and nitrogen‐doped porous carbon architecture in the shell (Ni@NC) for the efficient electroreduction of NO to ammonia (NH_3_) is reported. In Ni@NC, the NC prevents the dissolution of Ni nanoparticles and ensures the long‐term stability of the catalyst. The Ni nanoparticles involve in the catalytic reduction of NO to NH_3_ during electrolysis. As a result, the Ni@NC achieves a faradaic efficiency (FE) of 72.3% at 0.16 *V*_RHE_. The full‐cell electrolyzer is constructed by coupling Ni@NC as cathode for NORR and RuO_2_ as an anode for oxygen evolution reaction (OER), which delivers a stable performance over 20 cycles at 1.5 V. While integrating this setup with a PV‐electrolyzer cell, and it demonstrates an appreciable FE of >50%. Thus, the results exemplify that the core–shell catalyst based electrolyzer is a promising approach for the stable NO to NH_3_ electroconversion.

## Graphical abstract

Drastically reduced overpotential for NO to NH_3_ electroconversion on a core–shell electrocatalyst enables an energy‐efficient NORR in a PV‐assisted NO full‐cell electrolyzer. The carbon shell in the catalyst protects the Ni core from dissolution, thereby promoting better selectivity toward NH_3_ electrosynthesis in acidic medium.

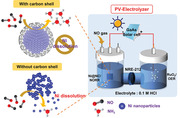

## 1 Introduction

**Figure 1:**
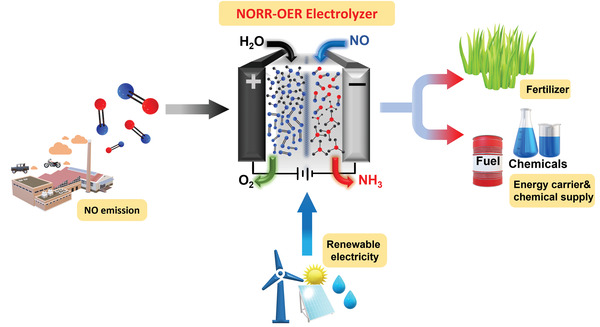
Schematic illustration of the strategies involved in the electroconversion of air pollutant NO to NH_3_ via electrolyzer powered by renewable electricity. The produced NH_3_ can be used as the feedstock of fertilizers, chemical supply, and energy carriers.

Nitric oxide (NO) is one of the major air pollutants released during the energy harvesting from fossil fuel combustion in thermal power plants, leads to increased air pollution,^[^ ^1^ ^]^ and causes serious health issues.^[^ ^2^ ^]^ High temperature ammonia‐selective catalytic reactor (NH_3_‐SCR) is generally used to abate the NO by converting it into harmless nitrogen (N_2_) gas which is of no chemical use.^[^ ^3^, ^4^ ^]^ Interestingly, the thermodynamically feasible electrochemical NO reduction reaction (NORR) to value‐added chemicals such as NH_3_^[^ ^5^ ^]^ and hydroxylamine (NH_2_OH)^[^ ^6^ ^]^ has been studied recently owing to its zero‐carbon emission pathway. In particular, NH_3_ production is considered a potential alternative to N_2_ as a source to produce NH_3_ through the energy‐intensive Haber–Bosch process operating under high temperatures (400–500 °C) and pressure (10–30 MPa).^[^ ^7^ ^]^ Moreover, the polar nature and less bonding energy of NO molecule than N_2_ (N=O 607 kJ mol^−1^, N≡N 941 kJ mol^−1^ at 25 °C) offers low activation energy and favors energy‐efficient electrolysis.^[^ ^8^ ^]^ In addition, NH_3_ has a versatile application in agriculture, pharmaceuticals, plastic industries, dye manufacturing, and, most notably, carbon‐free hydrogen energy carrier.^[^ ^9^ ^]^ Therefore, electrochemically utilizing NO for NH_3_ production, powered by renewable electricity, could be a welcoming approach that efficiently abates nitric oxide's air pollutant that brings the nitrogen cycle back into balance, as illustrated in **Figure** **1**.

Noble metals (such as Pt, Ru, Pd, and Ir) were used as an electrocatalyst to accelerate the reaction kinetics of the electroreduction of NO.^[^ ^10^ ^]^ However, the low availability, high cost, and commercial credibility are major drawbacks to hinder the extensive utilization of noble metals in NORR. Recently, transition metals^[^ ^5^, ^11^, ^12^ ^]^ and transition metal‐based single atoms,^[^ ^6^, ^13^ ^]^ phosphides,^[^ ^5^ ^]^ oxides,^[^ ^3^, ^14^ ^]^ and chalcogenide^[^ ^15^ ^]^ catalysts have been explored in dilute (10% NO) NORR to produce NH_2_OH and NH_3_. Nevertheless, it remains a pitfall to acquire a stable electrocatalyst that probes the electroconversion of concentrated NO gas to NH_3_ with low overpotential. For instance, Long et al. reported the improved NORR using Cu foam and produced unprecedented NH_3_.^[^ ^11^ ^]^ However, the driven electrolysis at high overvoltage (−0.9 V versus RHE) makes the process energetically disadvantageous. The solubility of NO in an aqueous electrolyte was increased by including iron (II) sodium benzoate complex, and NH_3_ was produced with ≈93% faradaic efficiency (FE). Although this approach is promising, it retards the practicability of attaining similar activity in gas phase NO electrolysis.^[^ ^15^ ^]^ The gas diffusion electrode (GDE) was employed as a catalyst substrate that offers a reduced gas diffusion pathway (≈50 nm), enhancing the mass transport of NO reactants.^[^ ^16^ ^]^ However, the feasibility of high content NO as source gas (99.9% NO) and securing the stability of the electrocatalyst for practical application is still under investigation. Unlike the wide pH range (2–12) typically employed for half‐cell studies, a strong acidic electrolyte is required for the device‐level NH_3_ production (e.g., the NORR‐oxygen evolution reaction [OER] electrolyzer) based on proton exchange membrane technology. Moreover, Cheon et al. reported the dissolution of the metal catalyst during electrolysis leads to catalyst deactivation in acid medium.^[^ ^16^ ^]^ Therefore, to overcome the drawbacks, for example, ensuring catalyst stability in an acidic environment, consuming concentrated NO as feed gas in electrolysis and NH_3_ selectivity at low overpotential could be achieved by employing structural engineering to tune the catalyst to increase its commercial value. Hence, the active metal site should be protected from direct contact with acidic NO molecules and harsh electrolytes without affecting its activity. Some of the strategies include: i) optimizing the framework/composition of active materials to increase more active sites; ii) tuning electronic properties to enhance the binding energy by doping heteroatoms; and iii) structural engineering of materials to boost the catalytic surface area.^[^ ^17^ ^]^ In this aspect, the metal‐rich carbon composites with mesoporous structures are a promising candidate since carbon material is anti‐corrosive to acids than most transition metals.^[^ ^18^ ^]^

Notably, a thin layer of carbon‐wrapped metal catalysts, that is, core–shell materials, is recognized to have excellent electrocatalytic activity and stability in robust operating conditions.^[^ ^19^ ^]^ The carbon shell allows fast mass permeability, a large area of exposed active metal surfaces, superior electron transfer during the reactions and its insolubility in most solvents, which makes them tremendously attractive for heterogeneous catalysis. Most importantly, the active core is protected by the carbon network, which ensures the high stability of the electrocatalyst compared to the bare metal nanoparticles. Prussian blue analog (PBA) was proven to be a good precursor to synthesize  the metal‐rich, thinner shell of nitrogen‐doped carbon electrocatalysts, which has been extensively studied in various electrocatalytic applications.^[^ ^18^, ^20^ ^]^

Inspired by the catalytic hydrogenation property of Ni (e.g., Raney nickel) in industrial processes, we have prepared a PBA‐derived, N‐doped porous nanocarbon encapsulated Ni nanoparticles (Ni@NC) as a core–shell electrode to prevent the dissolution of active Ni during the NORR. In the Ni@NC, the metal nanoparticles were coated with the porous, conducting NC network that facilitates ionic and gaseous transport.^[^ ^21^ ^]^ The nature of the active site and the role of the NC layer of the complex core–shell catalyst were revealed from the control experiments. Under the 100% NO gas‐saturated electrolyte, the Ni@NC‐3 has shown an onset potential of ≈0.4 *V*_RHE_ for NH_3_ formation and attained the maximum NH_3_ FE (FE_NH3_) of 72.3 ± 1.3% at 0.16 *V*_RHE_. The corresponding overpotential is 550 mV, among the lowest overpotentials reported to date in the production of NH_3_ from NO. Rational integration of such an electroreduction process with solar energy provides the prospect of utilizing air pollutants and sunlight for sustainable NH_3_ production. However, no reports demonstrate solar energy‐assisted NH_3_ synthesis using NO. Besides full‐cell study, for the first time, the idea of solar‐to‐fuel conversion was utilized in NORR in which a solar energy‐assisted NORR‐OER electrolyzer was constructed and achieved about 1.7% and >50% of solar‐to‐NH_3_ efficiency and FE_NH3_, respectively. These approaches open a different avenue for developing durable materials for NO to NH_3_ electroconversion and other energy conversion devices assisted by renewable electricity.

## 2 Results and Discussion

**Figure 2:**
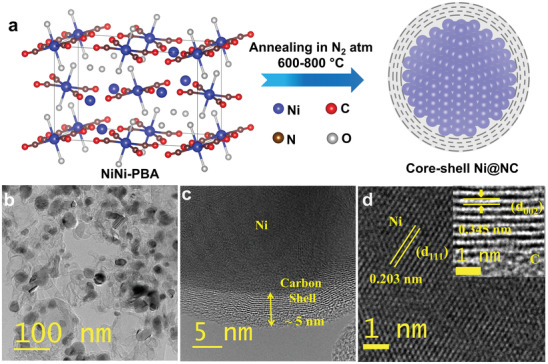
Synthetic route and morphological characterization of the core–shell Ni@NC catalyst. a) Schematic synthesis route of Ni@NC from NiNi‐PBA. b) TEM image. c,d) HR‐TEM images, (d) inset shows d‐spacing of the carbon shell of the Ni@NC‐3 catalyst.

The core–shell Ni@NC catalyst was synthesized by one‐step pyrolysis of Ni[Ni(CN)_4_] PBA (NiNi‐PBA) containing nickel, carbon, and nitrogen atoms in the temperature range of 600–800 °C under an inert atmosphere (see details in the Experimental Section), as shown in **Figure** **2**a. The formation of NiNi‐PBA was confirmed by the FE‐SEM and XRD analysis (Figure S1, Supporting Information). Ni cations from the analog will be reduced to form Ni nanoparticles. The CN— groups as linkers tend to form N‐doped porous carbon layers encapsulated in the nanoparticles during the high‐temperature pyrolysis process. Indeed, the porous nature thus permits reactants to access the Ni surface. More importantly, the in situ formed NC layers protect the nanoparticle core from corrosion, thus ensuring the catalyst's durability under vigorous reaction conditions.

Interestingly, TEM images of Ni@NC‐3 catalyst reveal a core–shell structure composed of dark spherical particles at the core and layered carbon as a shell surrounding the nanoparticles (Figure 2b). It shows the broad particle size distribution and size of the Ni particles which varied from 15 to 50 nm, with an average diameter of 25 nm. In addition, core–shell particles were interconnected by a smooth carbon matrix.

The high‐resolution TEM (HR‐TEM) reveals the single Ni particle surrounded by several layers of carbon (Figure 2c). The thickness of the carbon layer was found to be ≈5 nm, which consists of orderly stacked layers with a d‐spacing of 0.345 nm, corresponding to the sp^2^ carbon interplanar distance.^[^ ^7a^ ^]^ Figure 2d shows the Ni interatomic lattice spacing of 0.203 nm corresponding to the (111) plane of Ni, which agrees with the XRD results. The TEM images of Ni@NC‐1 and 2 catalysts show the same core–shell structures with carbon stacking at ≈2 and ≈3 nm, respectively (Figure S2, Supporting Information). Hence, the low annealing temperature leads to the formation of thinner graphitic carbon layers. The TEM elemental mapping exhibits the uniform distribution of Ni at the core, whereas the carbon and a trace amount of nitrogen form the shell in the Ni@NC‐3 (Figure S3, Supporting Information). Figure S4, Supporting Information, confirms the formation of Ni@NC with two different crystal structures: face centered cubic (FCC) and hexagonal close packing (HCP) of Ni nanoparticles. The XRD analysis implies that the crystallinity of the nanostructures increases with annealing temperature from 600–800 °C. To further evaluate the nature of the carbon shell and its structural defects, the Raman spectra were measured and are given in Figure S5, Supporting Information. The intensity ratios of D‐ and G‐band (*I*_D_/*I*_G_) of the samples decreased with the annealing temperature. Among the catalysts, Ni@NC‐3 shows the lowest *I*_D_/*I*_G_ ratio of 0.94, implying the presence of ordered carbon layers with a high graphitization degree. Thermogravimetric analysis (TGA) was employed to study the thermal stability of the NiNi‐PBA precursor under an inert atmosphere (Figure S6, Supporting Information). After 510 °C, the complex is stable, and only a slight decrease in the weight corresponds to the removal of nitrogen content, which coincides with CHNS results (Table S1, Supporting Information).

The X‐ray photoelectron spectroscopy (XPS) was conducted to examine the chemical nature of the core–shell catalysts, and the survey spectrum confirms the presence of nickel, carbon and nitrogen (Figure S7, Supporting Information). As seen from Figure S8g, Supporting Information, the deconvoluted Ni 2p spectra of the Ni@NC‐3 reveal two Ni 2p^3/2^ and Ni 2p^1/2^ peaks because of spin‐orbit coupling. The 852.7 and 869.6 eV peaks correspond to the Ni(0) species.^[^ ^19^ ^]^ In addition, the presence of two oxidized nickel peaks (Ni^+2^) at 855.1 and 872.5 eV were observed due to the surface oxidation of the catalyst.^[^ ^19^ ^]^ Also, their satellite peaks were observed at higher binding energies like 861.3 and 879.6 eV.^[^ ^19^ ^]^ Figure S8h, Supporting Information, shows the C 1s high resolution spectra, and there are four different binding peaks at 284.5, 285.2, 286.5 and 288.8 eV, revealed the presence of C—C, C—N, C—O and O=C—O bonds, respectively.^[^ ^22^ ^]^ The presence of the C—N bond confirms the occurrence of doped N in the carbon framework. Similarly, high‐resolution N 1s spectra was deconvoluted into three peaks at 398.8, 400.8 and 405.1 eV, corresponding to pyridinic‐N, Graphitic‐N and N—O bond, respectively (Figure S8i, Supporting Information).^[^ ^22^ ^]^ Moreover, the intensity and peak area of the graphitic‐N increased gradually with increasing pyrolyzing temperature, further confirming the high graphitization degree of the Ni@NC‐3. In addition, similar high‐resolution XPS spectra were obtained for the remaining Ni@NC catalysts, as shown in Figure S8, Supporting Information. These results confirmed the formation of core–shell Ni@NC catalysts with no other impurities.

### 2.1 Electrocatalytic Reduction of NO to NH_3_ in Acid Medium

**Figure 3:**
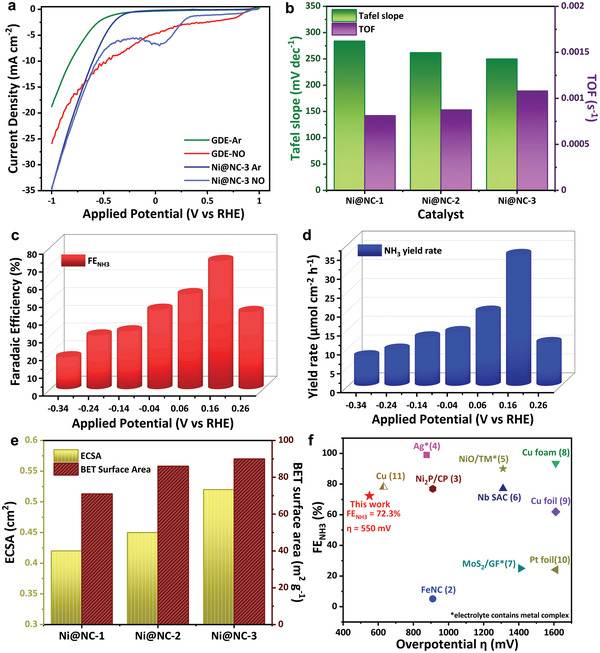
Electrochemical NORR performance of the Ni@NC‐3 catalyst in 0.1 м HCl. a) Polarization curves obtained on Ni@NC‐3 and GDE in Ar and NO‐saturated medium. b) Tafel slope and TOF comparison of Ni@NC catalysts. c) FE_NH3_ of Ni@NC‐3 at different potentials and d) the corresponding potential dependent ammonia yield rates. e) ECSA and BET surface area comparison of Ni@NC catalysts. f) Comparison of FE_NH3_ and corresponding overpotential of Ni@NC‐3 with recent NORR reports. The number in the brackets corresponds to the reference (Table S5, Supporting Information).

The electrocatalytic NORR tests were performed using an air‐tight H‐type electrochemical cell containing 0.1 м HCl separated by a Nafion membrane under ambient conditions. A three‐electrode setup was used in which catalysts coated on GDE as the working electrode, graphite rod as the counter electrode, and Ag/AgCl as the reference electrode was used to analyze the NORR study. To study the activity of Ni@NC catalysts, linear sweep voltammetry (LSV) analysis was performed at a scan rate of 5 mV s^−1^ in Ar and NO‐saturated electrolytes. In LSV, a different trend is observed due to the different mechanistic pathways followed by the Ni@NC‐3 and bare GDE during NORR (**Figure** **3**a). The Ni@NC‐3 catalyst displays no characteristic reduction until −0.2 *V*_RHE_ in Ar medium. However, a steep increase in the current density beyond −0.2 *V*_RHE_ was observed, corresponding to the hydrogen evolution reaction (HER) taken as background. In contrast, under NO‐saturated medium, dissolved NO in the electrolyte had started to consume at ≈1 *V*_RHE_, showing a small reduction current, followed by a drastic increase in the current density, and presented a distinct plateau below 0.1 *V*_RHE_. This could be explained by the fact that, from 0.4 to 0.1 *V*_RHE_, the reaction is kinetically controlled rather than by diffusion of active species, and below 0.1 *V*_RHE_, the NORR current is mediated by the mass transport pathway. Thus, the reduction peak at ≈0.1 *V*_RHE_ corresponds to the maximum diffusion‐limiting current for the formation of NH_3_ since the electroreduction of NO to NH_3_ in aqueous media is a diffusion‐controlled reaction. The trace amount of current monitored in the high potential <1 V_RHE_ exclusively ascribed to gaseous products such as N_2_ and N_2_O (E^0^ = 1.68 and 1.59 *V*_RHE_, respectively),^[^ ^16^, ^23^ ^]^ since there is no possibility of other NO products in this potential range. The polarization curves of all the core–shell Ni@NC catalysts synthesized at different annealing temperatures were evaluated (Figure S9, Supporting Information).

It suggested that the catalyst synthesized at 800 °C shows a high current density of ≈7 mA cm^−2^ with a distinct plateau at low potentials than other catalysts, which could be expected due to the exposure of more active centers, as evidenced by the large electrochemical active surface area (ECSA) (Figure 3e and Figure S10, Supporting Information). Furthermore, the Randles–Sevcik equation was employed to calculate the number of electrons transferred during the reduction reaction (Figure S11, Supporting Information). The obtained value (*n* = 4.83) indicates that the NORR pathway was mainly accompanied by five‐electron transfer in this region. Tafel slope values were extracted from the polarization curve, and the values were 285, 262 and 250 mV dec^−1^ for core–shell Ni@NC‐1, 2 and 3, respectively (Figure 3b and Figure S12, Supporting Information). Ni@NC‐3 shows a smaller Tafel slope of all catalysts, suggesting enhanced charge‐transfer kinetics. Furthermore, turnover frequency (TOF) was calculated based on the Ni metal obtained from ICP‐OES (see details in Note S1 and Table S4, Supporting Information), and the results are summarized in Figure 3b. At 0.16 *V*_RHE_, the observed TOF values were 0.000832, 0.000874 and 0.00104 s^−1^, respectively, for Ni@NC‐1, 2 and 3 catalysts, which revealed that Ni@NC‐3 catalyst possessed a larger TOF value because of higher intrinsic electrochemical activity. Thus, a smaller Tafel slope and higher TOF exhibited by the Ni@NC‐3 catalyst were considered the best catalytic activity toward NO electroreduction.

Furthermore, to examine the potential dependent product yield and FE_NH3_, chronoamperometry (CA) experiments for all the Ni@NC catalysts were carried out between the potential range of 0.3 and −0.4 *V*_RHE_ for 1 h. After electrolysis, an adequate amount of electrolyte was taken to quantify the products. The indophenol blue method and ^1^H NMR were used to quantify NH_3_ concentration (Figures S14 and S15, Supporting Information, respectively). Other products such as NH_2_OH and N_2_H_4_ were also examined using corresponding colorimetric methods (Figure S14, Supporting Information). Possible gaseous products such as H_2_, N_2_, and N_2_O were not considered during the entire NORR electrolysis. From Figure 3c,d, NH_3_ formation starts at 0.26 *V*_RHE_ for Ni@NC‐3 catalyst, shows a volcano‐type trend, and reaches the least FE_NH3_ at −0.34 *V*_RHE_. The maximum FE_NH3_ of 72.3 ± 1.3% with a reasonably high NH_3_ yield rate of 34.6 ± 1.1 µmol cm^−2^ h^−1^ was achieved at 0.16 *V*_RHE_ and decreased gradually as the potential goes more negative due to the competitive NORR side reactions. Meanwhile, N_2_H_4_ and NH_2_OH were not produced in the entire potential window, confirmed by the absence of characteristic UV–vis absorbance at 455 and 705 nm, respectively, from the colorimetric results (Figure S16, Supporting Information). The tail gas from the H‐cell was introduced into the acid trap, and no significant absorbance was observed at 655 nm indicating that there is no formation of NH_3_ gas during NORR. The electrocatalytic performance of all core–shell Ni@NC catalysts was compared in terms of both NH_3_ yield and FE_NH3_ (Figures S16–S19, Supporting Information). It suggested that the Ni@NC‐3 shows a reasonably maximum FE_NH3_ at 0.16 *V*_RHE_. This could be attributed to the high surface area of graphitized carbon layers that boost the specific capacitance by allowing for more active sites for the adsorption of active species (Figure 3e and Table S2, Supporting Information). The high electrode roughness of Ni@NC‐3 reduces the boundary layer formed at the interface (Table S3, Supporting Information).

Interestingly, the GDE shows a significant reduction current in NO‐medium, indicating that the carbon‐based materials are also active toward NORR (Figure 3a). However, a trace NH_3_ is observed, and most of the remaining current is due to the formation of gaseous products (such as N_2_ and N_2_O) from NO, confirmed by the evolution of gas bubbles in CA studies (Figure S20, Supporting Information), which is in good agreement with the previous reports.^[^ ^16^, ^24^ ^]^ Since GDE promotes the evolution of gaseous products, there is no distinct diffusion layer formation at the electrode–electrolyte interface, resulting in no prominent reduction peak as Ni@NC‐3. Moreover, the long‐term stability of the Ni@NC‐3 catalyst at 0.16 *V*_RHE_ for 24 h is steady, with negligible variation in the current density (Figure S21, Supporting Information). The FE_NH3_ of ≈72% was retained throughout the electrolysis. The amount of NH_3_ produced increased linearly with the reaction time, further emphasizing the ability of the Ni@NC‐3 for the stable NORR process. The highest FE_NH3_ of 72.3 ± 1.3% with a very low overpotential of 550 mV of our NORR catalyst outperformed the previously reported NORR results in the aqueous electrolyte (Figure 3f and Table S5, Supporting Information).

Moreover, various control experiments were conducted to identify the nitrogen source for the NH_3_ formation (Figure S22, Supporting Information). The UV–vis spectra showed no characteristic absorbance at 655 nm, when Ar was purged as a source gas, revealing that the nitrogen source originates from the NO feed gas only. Similarly, that no ammonia was identified when the cell was provided with open‐circuit voltage (OCV) also indicates that the reaction proceeds only under the application of external voltage. Also, the effect of catalyst loading was studied by varying the Ni@NC‐3 loading from 0.5 to 2 mg cm^−2^. The plot of ammonia yield and FE_NH3_ as a catalyst loading function derived from the potentiostatic electrolysis at 0.16 *V*_RHE_ (Figure S23, Supporting Information). The decreasing FE_NH3_ of the Ni@NC‐3 upon increasing the catalyst loading could be attributed to the thickened catalyst layer raising the charge transfer resistance.^[^ ^25^ ^]^

### 2.2 Elucidating the Nature of the Active Site and Identification of Reaction Pathway

**Figure 4:**
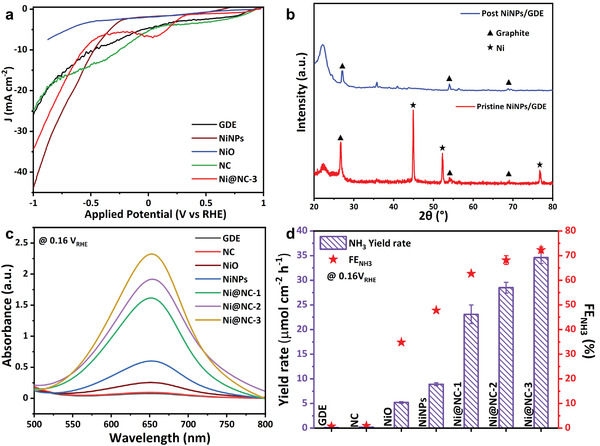
Electrochemical NORR performance of the control samples. a) Polarization curves of all control‐samples obtained in NO‐saturated medium. b) XRD analysis of the post and pristine NiNPs coated GDE after 1 h NORR study at 0.16 *V*_RHE_. c) UV–vis absorbance spectra of the analyte taken after 1 h NORR study at 0.16 *V*_RHE_ to quantify NH_3_. d) Comparison of NH_3_ yield and FE_NH3_ on various electrodes after 1 h NORR study at 0.16 *V*_RHE_.

The complex structural heterogeneity of the core–shell material impedes the basic identification of the nature of the NORR active site. The electrocatalytic activity of the core–shell catalyst depends on the synergistic effect of both the core and shell.^[^ ^26^ ^]^ Hence, we resorted to an indirect methodology, which involved the synthesis of individual counterparts of the core–shell catalyst, such as Ni nanoparticles (NiNPs), NC and NiO from NiNi‐PBA (see details in Experimental Section). The XRD analysis confirmed the formation of NiNPs, NC, and NiO (Figures S24–S26, Supporting Information). LSV measurements recorded for all the catalysts showed considerable NORR activity (**Figure** **4**a). To evaluate the current response, CA analysis was carried out at 0.16 *V*_RHE_.

The NiNPs catalyzed NORR gives a decent selectivity of 47.8% FE_NH3_ with NH_3_ yield rate of 8.9 µmol cm^−2^ h^−1^. As a crucial step, the in‐depth observation of the post NiNPs coated GDE revealed the dissolution of NiNPs from the electrode surface during NORR. The XRD analysis of the NiNPs coated GDE, taken before and after the electrolysis, showed no distinct Ni peaks (Figure 4b). It suggests the complete dissolution of NiNPs from the electrode, and in the further electrolysis, only bare GDE participates in the NORR, changing its selectivity toward other products such as N_2_, N_2_O and H_2_ (Figure S20a, Supporting Information). In contrast, the NC sample showed a relatively high cathodic current but yielded only 0.5 µmol cm^−2^ h^−1^ of NH_3_ (Figure 4c). We speculate the huge current is due to the N‐doped carbon matrix favoring the dimerization of NO molecules. Hence, the poor FE_NH3_ of NC may be assigned to the formation of gaseous nitrogen dimer products from NO, which was already confirmed from the carbon‐coated GDE experiments. In the core–shell Ni@NC catalyst, the carbon shell gives structural stability to the encapsulated NiNPs and provides a high surface area that paves the way to access abundant active sites. The doped‐nitrogen on the carbon framework highly polarizes the surface and boosts the adsorption energy for the incoming reactant molecules compared to bare NiNPs.^[^ ^27^ ^]^ In addition, the NC shell accelerates the electron transfer and facilitates the rapid diffusion of reactants to the active Ni centers to reduce it into NH_3_, emphasizing the high selectivity nature of the core–shell catalyst.

The chemical composition analysis reveals the presence of both Ni(0) and Ni(+2) states in Ni@NC‐3 (Figure S8, Supporting Information). To clarify the NORR activity contribution from Ni(+2), the synthesized NiO catalyst is subject to NORR and we obtained a polarization curve as shown in Figure 4a. When compared to Ni@NC‐3 catalyst, NiO exhibits poor catalytic activity toward NORR. Further CA analysis was conducted to examine the selectivity of NiO toward NH_3_ synthesis (Figure S27b, Supporting Information). The NH_3_ yield rate and FE_NH3_ of NiO are extremely low compared to the Ni@NC‐3 (Figure S27c,d, Supporting Information). These findings demonstrate that the NiO may also involve in NORR, but its contribution is relatively low compared to the Ni@NC‐3. The NORR performance of all control samples in terms of NH_3_ yield and FE_NH3_ shows that the Ni@NC‐3 results in the highest activity compared to their individual counterparts (Figure 4c,d). Also, pure NiNPs catalyst exhibits inferior stability in the acid medium, again emphasizing the importance of carbon shell. As the thickness of the carbon shell increases, the electroconversion of NO to NH_3_ increases due to its high mesoporous nature. Therefore, the synergistic effect of nickel and the nitrogen‐doped carbon nanostructures is responsible for the highest NORR performance in the Ni@NC‐3 catalyst.

The electrochemical NORR process involves multiple reaction pathways, each of which leads to different products depending on the applied potential and nature of the catalyst (Equations ((1), (2), (3), (4))).

(1) $$\mathrm{NO}+3\left(H^{+}+e^{-}\right)\to {\mathrm{NH}}_{2}\mathrm{OH}\left(E^{0}=0.38V\right)$$

(2) $$\mathrm{NO}+5\left(H^{+}+e^{-}\right)\to {\mathrm{NH}}_{3}+H_{2}O\left(E^{0}=0.71V\right)$$

(3) $$2\mathrm{NO}+2\left(H^{+}+e^{-}\right)\to N_{2}O+H_{2}O\left(E^{0}=1.59V\right)$$

(4) $$2\mathrm{NO}+4\left(H^{+}+e^{-}\right)\to N_{2}+2H_{2}O\left(E^{0}=1.68V\right)$$

The entire reaction to form NH_3_ and H_2_O from NO involves five protonation steps. The NO adsorption on the catalyst can be followed by N‐end, O‐end, or side‐on configuration pathway.^[^ ^28^ ^]^ It has been reported that a similar Ni surface favors O‐end adsorption pattern, followed by either a distal or alternating pathway to produce NH_3_, as seen in nitrogen reduction reaction (NRR).^[^ ^5^ ^]^ Moreover, the distal pathway involves the stepwise proton–electron coupled reaction, while the alternative pathway follows NH_2_OH formation as an intermediate step in addition to hydrogenation to produce NH_3_.^[^ ^5^, ^7^ ^]^ Both pathways initiate with the first hydrogenation step (*NO + H^+^ + e^−^ → *NOH, *denotes the adsorption site), followed by the two different routes. It should be noted that it is experimentally difficult to decouple the underlying NORR pathway on Ni surface. However, an indirect approach was performed to identify the possible reaction pathway of the Ni@NC‐3 toward NORR. For that, the NH_2_OH was intentionally injected into the electrolyte, and the activity of the Ni@NC‐3 catalyst was evaluated over the wide NORR potential window. Since the Ni@NC‐3 catalyst has no electrochemical activity toward NH_2_OH, the polarization curves of Ni@NC‐3 in Ar medium or varied concentrations of NH_2_OH are almost the same (Figure S28a, Supporting Information). Furthermore, CA results at different potentials ensure that the concentration of NH_2_OH was the same before and after the study (Figure S28b,c, Supporting Information). On the other side, ^1^H NMR was analyzed for the catholyte, in which the characteristic triplet of NH_3_ (6.7–7.1 ppm) were not detected (Figure S28d, Supporting Information). It confirms that Ni has no binding affinity with NH_2_OH to produce NH_3_. These findings suggest that the Ni@NC‐3 possibly follows the O‐end configuration with distal pathway (*NO + H^+^ + e^−^ → *NOH; *NOH + H^+^ + e^−^ → *N + H_2_O; *N + H^+^ + e^−^ → *NH; *NH + H^+^ + e^−^ → *NH_2_; *NH_2_ + H^+^ + e^−^ → *NH_3_) to yield NH_3_ from NO.

### 2.3 Coupling NORR and OER in the Full‐Cell Electrolyzer

**Figure 5:**
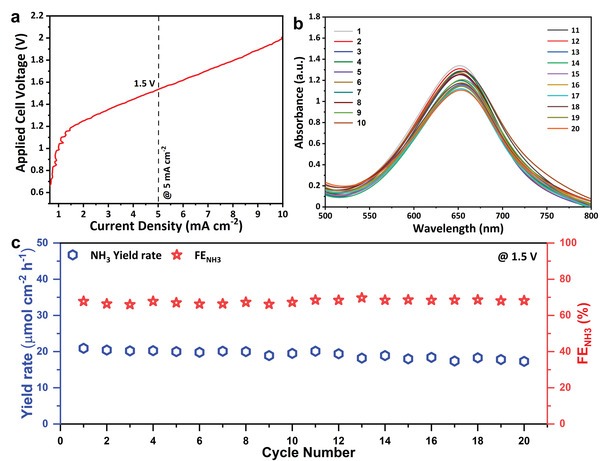
Full‐cell electrolysis by coupling Ni@NC‐3 for NORR and RuO_2_ for OER. a) Polarization curve for the full‐cell NORR‐OER electrolyzer. b) UV–vis absorbance spectra of the analyte taken every cycle after the NORR‐OER electrolysis to quantify NH_3_. c) Recycling durability test of the NORR‐OER electrolyzer at a cell voltage of 1.5 V under ambient conditions.

Encouraged by the efficient low overvoltage NORR electrolysis in half cell studies, we constructed a full‐cell by coupling NORR at the cathode and OER at the anode to evaluate its practical capability. Here, RuO_2_ was utilized as an OER electrocatalyst. Even though it is costly, RuO_2_ is valued for its excellent activity toward OER in acidic medium.^[^ ^29^ ^]^ Therefore, RuO_2_ was employed as a benchmark material to couple with Ni@NC‐3 NORR. At first, the polarization curve for RuO_2_ toward OER in 0.1 м HCl was measured using the three‐electrode setup (Figure S29, Supporting Information). RuO_2_ exhibited an overpotential of 200 and 370 mV to reach a benchmark current density of 1 and 10 mA cm^−2^, respectively. A full‐cell electrolyzer was constructed with a two‐chamber reactor, and a typical non‐IR compensated activity curve was generated (**Figure** **5**a). The electrolysis starts at ≈1.1 V (consistent with the combined half‐cell potential of NORR and OER). It requires only a 580 mV overvoltage from the thermodynamic NORR‐OER cell voltage (E°_cell_ = 0.52 V), which is good for enabling energy‐efficient NORR electrolysis. The cell voltage of 1.5 V is required to reach the benchmark of 5 mA cm^−2^ as depicted from the polarization curve, which was chosen to evaluate the cyclability of the full‐cell. The full‐cell electrolyzer can retain the FE_NH3_ of ≈68% for 20 cycles under the highly oxidizing NORR conditions. The corresponding CA curves are given in Figure S30, Supporting Information. The UV–vis absorbance spectra of the electrolyte‐stained with indophenol were used to estimate NH_3_ (Figure 5b). The average yield rate of NH_3_ was 20 µmol cm^−2^ h^−1^ (Figure 5c), and the energy efficiency (EE) of the cell was calculated to be ≈23%. The reasonably high FE_NH3_ and EE obtained in a full‐cell configuration intended the extensive utilization of solar energy to convert air pollutants into value‐added chemicals.

### 2.4 Electrocatalytic NORR Driven by a Solar Cell

**Figure 6:**
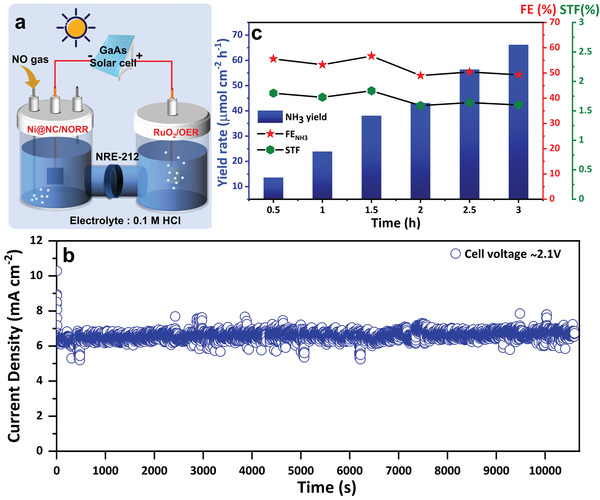
Solar energy‐assisted full‐cell NORR‐OER electrolysis. a) Schematic of PV‐electrolyzer system for solar energy‐assisted NH_3_ synthesis. b) Stable operating current obtained during the electrolysis for 3 h, and c) the corresponding time‐dependent NH_3_ yield rate, FE_NH3_ and STF efficiency.

The utilization of electricity generated from renewable energy sources such as solar is highly beneficial and paves a path for an end‐to‐end solution for the zero‐carbon environment. Accordingly, we used solar energy to drive the electrolysis that can directly produce NH_3_ from the air pollutant NO, as displayed in the schematic **Figure** **6**a. A commercial triple‐junction GaAs solar panel was employed to power the electrolysis illuminated with simulated AM 1.5 G solar light.

At first, the *j*–*V* curve of the solar panel was measured under diffuse light and 1 sun condition (Figure S31, Supporting Information). A similar H‐type cell reactor, consisting of Ni@NC‐3 as NORR cathode and RuO_2_ as OER anode, was used for the solar energy‐assisted electrolysis (Figure S32, Supporting Information). The *i*–*t* curve of the PV‐electrolyzer with an operating cell voltage of ≈2.1 V was measured for 3 h (Figure 6b). The electrolyte aliquots were taken periodically every 0.5 h to evaluate the ammonia produced in the catholyte chamber (Figure S33, Supporting Information). The FE_NH3_ of >50% was attained, which remained almost unaltered during the 3 h of CA study (Figure 6c). Additionally, the solar‐to‐fuel (STF) efficiency was calculated to be ≈1.7%, which is comparable to a laboratory‐scale setup with limited NO utilization.

### 2.5 Post NORR Study for a Ni@NC‐3 to Evaluate the Chemical and Morphological Features

After NORR studies, the characterization of the catalyst can provide information about the core–shell Ni@NC in terms of structural, morphological, and chemical nature. The presence of XRD peaks at 44.4°, 51.5° and 76.4° corresponds to (111), (200) and (220) facets of Ni(0) (JCPDS: 98‐005‐3808), and confirms the presence of Ni in Ni@NC‐3 after the cyclability test in the acidic electrolyte (Figure S34a, Supporting Information). Similarly, the XRD analysis of Ni@NC‐1, Ni@NC‐2, and NC before and after the electrolysis retains the pristine crystal structure (Figure S35, Supporting Information). The XPS was studied to evaluate the oxidation state of each element (Figure S34b–d, Supporting Information). The high‐resolution XPS spectra of Ni 2p and N 1s show a shift in binding energy compared to the pristine Ni@NC‐3. The partial oxidation of Ni(0) to Ni(+2) may be caused by the long‐term exposure of Ni@NC‐3 in the oxidizing environment during NO electrolysis. In addition, the shift in binding energy is due to the delocalization of unpaired electrons of Ni(+2) in the oxide layer.^[^ ^30^ ^]^ More interestingly, the retention of N 1s peaks in XPS spectra further ensures that NO is the only source for NH_3_ production in NORR rather than the N atom from the Ni@NC catalyst. Furthermore, the increased content of graphitic‐N after the stability test is proposed to be the graphitization of carbon layers during NORR, which facilitates stability to NC nanostructures.^[^ ^31^ ^]^ The post‐Raman analysis of Ni@NC‐3 shows that the ratio of *I*_D_/*I*_G_ decreased to 0.89, reveals the graphitization of carbon domains occurs during the stability test, which coincides with the XPS results (Figure S36, Supporting Information). Furthermore, the FE‐SEM and TEM analyses were carried out to study the morphology stability (Figure S37, Supporting Information). No significant change was observed in the morphology of Ni@NC‐3, thus encouraging the robustness of the core–shell nanostructures for prolonged electrolysis under corrosive reaction conditions. The above findings show that the core–shell electrocatalysts display superior chemical and morphological stabilities during NORR; hence, it could be employed as a potential catalyst in real‐time NO‐electrolyzer applications.

## 3 Conclusion

We have explored a low‐cost and scalable coprecipitation methodology to synthesize a highly‐graphitic N‐doped carbon shell encapsulated Ni nanoparticles for the stable and selective electroreduction of NO to NH_3_. By conducting a series of control tests, the active center of the NORR was discovered to be Ni in the core–shell Ni@NC. Also, the multilayer NC possesses an anti‐corrosive property which retards the demetalation of Ni, thereby ensuring stability during NORR. The Ni@NC‐3 electrode achieved a maximum FE_NH3_ of 72.3 ± 1.3% in the acid medium at 0.16 *V*_RHE_. Further, the two‐electrode NORR‐OER electrolysis was carried out at 1.5 V shows an excellent recycling stability for 20 cycles. With the help of detailed understanding in a full‐cell, PV‐electrolyzer cell was demonstrated and had shown remarkably >50% of FE_NH3_ with 1.7% of STF efficiency. The displayed performance of the electrolyzer paves a turning point toward the stable electroconversion of NO to value‐added chemicals. In particular, the excellent activity provided by the unique Ni@NC core–shell catalyst at low overvoltage could be a potential material to utilize in large‐scale solar‐driven NH_3_ generation.

## Conflict of Interest

The authors declare no conflict of interest.

## Supporting information

Supporting Information

## Data Availability

The data that support the findings of this study are available from the corresponding author upon reasonable request.

## References

[1] J. Rockström , W. Steffen , K. Noone , Å. Persson , F. S. Chapin , E. F. Lambin , T. M. Lenton , M. Scheffer , C. Folke , H. J. Schellnhuber , B. Nykvist , C. A. de Wit , T. Hughes , S. van der Leeuw , H. Rodhe , S. Sörlin , P. K. Snyder , R. Costanza , U. Svedin , M. Falkenmark , L. Karlberg , R. W. Corell , V. J. Fabry , J. Hansen , B. Walker , D. Liverman , K. Richardson , P. Crutzen , J. A. Foley , Nature 2009, 461, 472. 19779433 10.1038/461472a

[2] P. M. Vitousek , J. D. Aber , R. W. Howarth , G. E. Likens , P. A. Matson , D. W. Schindler , W. H. Schlesinger , D. G. Tilman , Ecol. Appl. 1997, 7, 737.

[3] a) L. Han , S. Cai , M. Gao , J.‐y. Hasegawa , P. Wang , J. Zhang , L. Shi , D. Zhang , Chem. Rev. 2019, 119, 10916; 31415159 10.1021/acs.chemrev.9b00202

[4] Y. Lin , J. Liang , H. Li , L. Zhang , T. Mou , T. Li , L. Yue , Y. Ji , Q. Liu , Y. Luo , N. Li , B. Tang , Q. Wu , M. S. Hamdy , D. Ma , X. Sun , Mater. Today Phys. 2022, 22, 100611.

[5] a) D. Kim , D. Shin , J. Heo , H. Lim , J.‐A. Lim , H. M. Jeong , B.‐S. Kim , I. Heo , I. Oh , B. Lee , M. Sharma , H. Lim , H. Kim , Y. Kwon , ACS Energy Lett. 2020, 5, 3647;

[6] a) D. H. Kim , S. Ringe , H. Kim , S. Kim , B. Kim , G. Bae , H.‐S. Oh , F. Jaouen , W. Kim , H. Kim , C. H. Choi , Nat. Commun. 2021, 12, 1856; 33767159 10.1038/s41467-021-22147-7 PMC7994811

[7] a) D. K. Yesudoss , H. Chun , B. Han , S. Shanmugam , Appl. Catal., B 2022, 304, 120938;

[8] Y.‐i. Kwon , S. K. Kim , Y. B. Kim , S. J. Son , G. D. Nam , H. J. Park , W.‐C. Cho , H. C. Yoon , J. H. Joo , ACS Energy Lett. 2021, 6, 4165.

[9] a) R. Schlögl , Angew. Chem., Int. Ed. 2003, 42, 2004; 10.1002/anie.200301553 12746811

[10] a) A. C. A. de Vooys , M. T. M. Koper , R. A. van Santen , J. A. R. van Veen , Electrochim. Acta 2001, 46, 923;

[11] J. Long , S. Chen , Y. Zhang , C. Guo , X. Fu , D. Deng , J. Xiao , Angew. Chem., Int. Ed. 2020, 59, 9711. 10.1002/anie.202002337 32189423

[12] T. Muthusamy , S. Sethuram Markandaraj , S. Shanmugam , J. Mater. Chem. A 2022, 10, 6470. 10.1002/advs.202201410 PMC9561790 35981872

[13] X. Peng , Y. Mi , H. Bao , Y. Liu , D. Qi , Y. Qiu , L. Zhuo , S. Zhao , J. Sun , X. Tang , J. Luo , X. Liu , Nano Energy 2020, 78, 105321.

[14] P. Liu , J. Liang , J. Wang , L. Zhang , J. Li , L. Yue , Y. Ren , T. Li , Y. Luo , N. Li , B. Tang , Q. Liu , A. M. Asiri , Q. Kong , X. Sun , Chem. Commun. 2021, 57, 13562. 10.1039/d1cc06113e 34842863

[15] a) L. Zhang , J. Liang , Y. Wang , T. Mou , Y. Lin , L. Yue , T. Li , Q. Liu , Y. Luo , N. Li , B. Tang , Y. Liu , S. Gao , A. A. Alshehri , X. Guo , D. Ma , X. Sun , Angew. Chem., Int. Ed. 2021, 60, 25263; 10.1002/anie.202110879 34519397

[16] a) M. J. Foral , S. H. Langer , Electrochim. Acta 1991, 36, 299;

[17] J. Ren , M. Antonietti , T.‐P. Fellinger , Adv. Energy Mater. 2015, 5, 1401660.

[18] a) Y. Feng , X.‐Y. Yu , U. Paik , Sci. Rep. 2016, 6, 34004; 27658968 10.1038/srep34004 PMC5034270

[19] a) A. Sivanantham , S. Shanmugam , ChemElectroChem 2018, 5, 1937;

[20] a) J. Su , Y. Yang , G. Xia , J. Chen , P. Jiang , Q. Chen , Nat. Commun. 2017, 8, 14969; 28440269 10.1038/ncomms14969 PMC5413983

[21] a) J.‐Y. Kim , D. Hong , J.‐C. Lee , H. G. Kim , S. Lee , S. Shin , B. Kim , H. Lee , M. Kim , J. Oh , G.‐D. Lee , D.‐H. Nam , Y.‐C. Joo , Nat. Commun. 2021, 12, 3765; 34155218 10.1038/s41467-021-24105-9 PMC8217160

[22] A. Sivanantham , P. Ganesan , L. Estevez , B. P. McGrail , R. K. Motkuri , S. Shanmugam , Adv. Energy Mater. 2018, 8, 1702838.

[23] H. Wan , A. Bagger , J. Rossmeisl , Angew. Chem., Int. Ed. 2021, 60, 21966. 10.1002/anie.202108575 34350689

[24] B. H. Ko , B. Hasa , H. Shin , Y. Zhao , F. Jiao , J. Am. Chem. Soc. 2022, 144, 1258. 35014265 10.1021/jacs.1c10535

[25] a) S. Anantharaj , S. Kundu , ACS Energy Lett. 2019, 4, 1260;

[26] S. Das , J. Pérez‐Ramírez , J. Gong , N. Dewangan , K. Hidajat , B. C. Gates , S. Kawi , Chem. Soc. Rev. 2020, 49, 2937. 32407432 10.1039/c9cs00713j

[27] M. B. Gawande , A. Goswami , T. Asefa , H. Guo , A. V. Biradar , D.‐L. Peng , R. Zboril , R. S. Varma , Chem. Soc. Rev. 2015, 44, 7540. 26288197 10.1039/c5cs00343a

[28] Y. Zang , Q. Wu , S. Wang , B. Huang , Y. Dai , Y. Ma , J. Phys. Chem. Lett. 2022, 13, 527. 35007068 10.1021/acs.jpclett.1c03938

[29] Y. Lin , Z. Tian , L. Zhang , J. Ma , Z. Jiang , B. J. Deibert , R. Ge , L. Chen , Nat. Commun. 2019, 10, 162. 30635581 10.1038/s41467-018-08144-3 PMC6329788

[30] S.‐F. Hung , Pure Appl. Chem. 2020, 92, 733.

[31] a) N. Zhou , N. Wang , Z. Wu , L. Li , Catalysts 2018, 8, 509;

