# Supplementary material for: Electrochemical Reduction of Nitric Oxide with 1.7% Solar‐to‐Ammonia Efficiency Over Nanostructured Core‐Shell Catalyst at Low Overpotentials
Source: Adv Sci (Weinh). 2022 Aug 18;9(29):2201410. doi: 10.1002/advs.202201410 (PMC9561790; doi:10.1002/advs.202201410)
Supplement: Supplementary file 1 — Supporting Information [file ADVS-9-2201410-s001.pdf]

Supporting Information

Electrochemical Reduction of Nitric Oxide with 1.7% Solar-to-Ammonia Efficiency Over Nanostructured Core-shell Catalyst at Low Overpotentials

*Sridhar Sethuram Markandaraj, Tamilselvan Muthusamy and Sangaraju Shanmugam**

Experimental Methods

Chemicals and materials: Nickel nitrate hexahydrate ($Ni(NO_3)_2.6H_2O$, Alfa aesar), Potassium tetracyanonickelate(II) hydrate ($K_2[Ni(CN)_4].H_2O$, Alfa Aesar), Iridium oxide (99%, IrO_2, Alfa aesar), Hydrochloric acid (HCl 37%, Sigma Aldrich), Sodium nitroprusside (99%, $Na_2[Fe(CN)_5NO]_2H_2O$, Sigma Aldrich), Sodium hypochlorite (NaOCl, reagent grade, chlorine 10-15%, Sigma Aldrich), Salicylic acid (99%, $C_7H_6O_3$, Samchun Chemicals), Trisodium citrate dihydrate (99%, $Na_3C_6H_5O_7$, Junsei chemicals), Ammonium chloride (99%, NH_4Cl, Samchun chemicals), Sodium hydroxide (NaOH, Samchun chemicals) were purchased and used without any further purifications. Nafion perfluorinated resin solution (Sigma Aldrich), Isopropyl alcohol (extra pure, C_3H_8O, OCI), Deionized water (DIW) were used as the solvents. Nafion membrane (NRE-212, Fuel cell store), Gas diffusion layer (GDL; Sigracet 39BC, SGL) composed of carbon layer with a microporous layer, NO gas (99.9%, Korea Standard Gas) were used as received.

Synthesis of Prussian blue analog (PBA): The Prussian blue analog, $Ni[Ni(CN)_4].nH_2O$, was synthesized according to our previous publication.[1] In brief, $Ni(NO_3)_2$ (100 mL, 0.1 M) was taken and stirred for 15 minutes until a clear solution was obtained. To the above solution, $K_2[Ni(CN)_4]$ (100 mL, 0.18 M) was added dropwise while the solution turned into sky blue color, indicating the nucleation of $Ni[Ni(CN)_4]_2.nH_2O$. For the complete interaction and formation of the product, the as-settled powder was aged in mother liquid for 24 h. Then, the solid powder was washed several times with deionized water to eliminate the impurities,

followed by ethanol washing. The collected solid powder was dried in air oven at 60 °C overnight and named as NiNi-PBA.

Preparation of core-shell electrocatalysts: To obtain the core-shell particles, the as-synthesized NiNi-PBA was annealed under N_2 atmosphere for 3 h. To evaluate the influence of carbon and nitrogen in the catalyst, the precursors were annealed at different temperatures such as 600, 700, 800 °C with a heating rate of 2 °C/min. The products obtained were named as Ni@NC-1, Ni@NC-2, and Ni@NC-3, respectively.

Preparation of nickel electrocatalyst: To study the importance of nitrogen-doped carbon shell, only metal catalysts were prepared from the NiNi-PBA. To synthesize nickel nanoparticles, the precursor was heated in air medium at 850 °C, followed by annealing at 500 °C under an Ar (10% H_2) gas flow for 3 h.

Preparation of NiO electrocatalyst: The NiO electrocatalyst was prepared by heating the NiNi-PBA at 600 °C for 3 h in air atmosphere.

Preparation of NC electrocatalyst: For comparison, NC catalyst was prepared by the acid leaching method. At the first adequate amount of Ni@NC catalyst was added to concentrated H_2SO_4 (50 mL) and stirred for two days. After filtering, the residue was added to concentrated HCl (50 mL) again and stirred for two days. The resultant mixture was washed with deionized water and ethanol multiple times, denoted as NC catalyst.

Characterizations: The X-ray Diffraction (XRD) analysis was studied with a scanning rate of 2° min^{-1} in the 2θ range of 10-90° using Miniflex 600, Rigaku diffractometer having Cu Kα radiation (λ = 1.5418 Å). Field Emission Scanning Electron Microscope (FESEM) imaging was evaluated using a Hitachi S-4800. The High-Resolution Transmission Electron Microscopy (HR-TEM) analysis was performed by a Hitachi HF3600 microscope. X-ray photoelectron spectroscopic (XPS) analysis was done by Thermo Scientific ESCALAB 250Xi, having micro-focused monochromator (Al Kα XPS) apparatus. The UV-Visible absorption spectra of the indicator-stained analytes were acquired using a Cary 5000 UV-VIS-NIR, Agilent Technologies. ^{1}H NMR Spectrum was completed using a Bruker, AVANCE III 400 Fourier-Transform Nuclear Magnetic Resonance Spectrometry (400 MHz, DMSO-d_6, δ: 2.5). Raman Spectroscopic study was evaluated by Thermo Scientific, Nicolet Almega XR.

Thermogravimetric Analysis (TGA) was carried out by TGA/Auto Q500 under an inert atmosphere. The CHNS analysis was carried out using an elemental analyzer (Vario MICRI cube) setup to quantify carbon and nitrogen in the catalysts. The porous nature of all the catalysts as characterized by BET, Micromeritics ASAP 2020. The Bruner-Emmett-Teller method was used to calculate the surface area and pore size distribution. All solar-driven electrolysis experiments were carried out using a conventional solar panel (GaAs thin-film solar cell) powered by a solar simulator (K3300ELX/ Cytec Korea) to illuminate simulated sunlight (1 Sun, AM 1.5 G). The current consumed during electrolysis was monitored by a 2636A-digital source meter (Keithley).

Electrochemical characterizations: The entire NORR study was conducted using a multi-channel potentiostat (Biologic, VSP) and air-tight H-type electrochemical cell separated by the Nafion-212 membrane. The Nafion membrane was pretreated with H_2O_2/ DIW mixture (1:5) at 100 °C for 1 h to remove the organic impurities, followed by subsequent rinsing and boiling in DIW. Then, the membrane was boiled in H_2SO_4 (0.5 M) for 1 h for protonation and finally washed with DIW. The catalyst-loaded GDE, graphite rod, and Ag/AgCl (saturated KCl electrolyte) were used as working, counter, and reference electrodes. Before starting each electrolysis, the ohmic resistance between the working electrode and the reference electrode was estimated using electrochemical impedance spectroscopy (EIS) between 200 kHz and 1 Hz with an amplitude of 10 mV. The resistance value was then determined by the intersection of the curve with the Real (Ω) axis in the Nyquist plot. iR correction was performed after electrolysis for all measurements. Every measurement was performed on a freshly prepared electrode. Current densities were calculated based on the catalyst-covered geometric area of the working electrode. The polarization curves were obtained at a scan rate of 5 mV s^{-1} and all the potential values were converted to RHE scale using the Nernst relation ($E_{RHE} = E_{WE} + E°_{Ag/AgCl} + 0.059$ pH, $E°_{Ag/AgCl} = 0.197$ V). All the experiments were carried out at room temperature (~25°C). To carry out electrolysis, the two chambers of the H-cell were filled with HCl (0.1 M). To ensure the complete elimination of dissolved oxygen, the cathodic compartment was purged with high purity Ar gas for at least 1 h. For the entire study, the high concentrate NO (100 %) was used as a source gas and purged (at rate of 1 sccm) with a suitable sparger in the cathode compartment. The headspace was covered by Ar flow to prevent NO_2 formation and O_2 dissolution. To capture the possible ammonia gas product, the tail gas of the catholyte chamber was trapped in an acidic HCl solution. After the electrolysis, the excess dissolved NO gas was removed by purging the Ar gas. For the full-cell

experiments, catalyst-coated GDE was used as NORR electrocatalyst, and RuO_2 (20 wt%) was used as the OER electrocatalyst with areal loading of 1 mg cm^{-2}. NORR-OER electrolysis was performed in the same two-compartment cell controlled by the potentiostat in the two-electrode configuration or powered by a conventional solar panel illuminated with simulated sunlight.

Electrochemical active surface area: Electrochemical active surface area (ECSA) of all catalysts was determined from the double-layer capacitance (C_{dl}) in a non-Faradic region using a typical cyclic voltammetry (CV) method. The double-layer current (i) is equal to the product of the scan rate (v) and C_{dl} ($i = vC_{dl}$), which is expected to be linearly proportional to the ECSA of the electrode. The C_{dl} was determined as half of the slope by plotting the capacitive currents (ΔJ, $J_{anodic} - J_{cathodic}/2$) versus v. The difference between the anodic and cathodic current was obtained at the potential of 0.66 V_{RHE}. Finally, the ECSA was estimated by dividing the C_{dl} by the specific capacitance (ECSA=C_{dl}/C_s, here the C_s value of 0.035 mF cm^{-2} for HCl (0.1 M) was used, based on the reported average C_s of Ni-based catalyst in acidic solution).

Electrode fabrication: The spray coating technique was applied to spray catalyst ink on GDE (1 x 1 cm^2) until 1 mg cm^{-2} loading was achieved. The catalyst ink was prepared by dispersing 5 mg of catalyst in IPA (500 μL), DIW (100 μL), and 5 wt% Nafion solution (50 μL). The resultant solution is sonicated for 45 min to form a homogeneous catalyst ink.

Product quantification: Possible gaseous products such as H_2, N_2, N_2O were not considered during the entire NORR electrolysis. However, tail gas from the cell was introduced into the acid trap to monitor gaseous NH_3. NH_3 was quantified using the indophenol blue method and ^1H NMR. NH_2OH and N_2H_4 were estimated using colorimetric methods. The NH_3 was determined spectroscopically using indophenol blue method.[2] After 1 h of electrolysis, 2 mL of analyte was mixed with Solution A (2 mL), Solution B (1 mL), and Solution C (200 μL) (Solution A: NaOH (1 M) containing salicylic acid (5 wt.%), trisodium citrate dihydrate (5 wt.%); solution B: sodium hypochlorite (0.05 M); Solution C: 1 wt.% Sodium nitroprusside). After 1 h incubation at dark, absorption at 655 nm was taken using UV-vis spectrophotometer to calculate the ammonia yield. Similarly, ammonium chloride of known concentration was dissolved in HCl (0.1 M) to obtain a standard calibration plot. To quantify ammonia using ^1H NMR, analyte (400 μL) was blended with H_2SO_4 (50 μL, 4 M) and DMSO-

d_6 (50 μL). Maleic acid of known concentration was added as an internal standard to obtain quantifiable data. At the same time, the ammonia was calculated by integrating the triplet with respect to the standard maleic acid peak (6.25 δ). Similarly, ^{1}H NMR spectra of electrolyte-containing known concentrations of ammonium chloride were derived to draw a linear plot. It is important to note that the acquisition with ordinarily applied pulse sequence (zg30) did not deliver any characteristic triplet peaks of ammonia except if the number of scans was higher (>512). This is because of dominating proton signals from water since it is an aqueous solvent. To eliminate this issue, sculpting pulse sequence (zgesgp) was employed along with the solvent suppression having a relaxation delay (d1) of 3 s and 256 scans during acquisition. The amount of hydrazine in the electrolyte was quantified by the Watt and Chrisp method.[3] At first, the color reagent was prepared by mixing para-(dimethylamino) benzaldehyde (5.99 g) in concentrated HCl (30 mL) and ethanol (300 mL). Analyte (2 mL) was diluted with DIW and blended with KOH (1 mL, 1 м), followed by adding color reagent (5 mL). After incubated in the dark for about 10 min, the absorbance at a wavelength of 455 nm was taken to calculate the amount of hydrazine formed. In the same way, the absorption-concentration curve was obtained by dissolving a known concentration of hydrazine hydrate in HCl (0.1 м) electrolyte. NH_2OH was quantified by a colorimetric method.[4] In brief, analyte (1 mL), phosphate buffer solution (1 mL, 0.05 м), DIW (0.8 mL), trichloroacetic acid (0.2 mL), 8-quinolinol (1 mL) were taken and swirled gently, followed by adding Na_2CO_3 (1 mL, 1 м). The resultant solution was shaken vigorously with a stopper; finally, the tube was kept in a boiling water bath for color development. Then, the solution blend was cooled at room temperature for 15 min, and the absorbance was taken at a wavelength of 705 nm to evaluate concentration. Following the same procedure, a calibration curve was established by testing a series of $NH_2OH.HCl$ solutions in the concentration range of 6-40 μм.

Equations used for the calculation:

The average yield rate (Y_p) of the product was calculated as follows:

$$Y_p = \frac{C_{product} \times V}{A \times t \times M_w}$$

Where Y_p is the products formation rate (μmol cm^{-2} h^{-1}), V is the total volume of electrolyte (mL), A is the electrode area (cm^2), t is time (h) for NORR, and M_w is the molar mass of the product (g mol^{-1}).

The Faradaic Efficiency (FE) can be calculated using the following formula:

$$\mathrm{FE} = \frac{\mathrm{n} \times \mathrm{F} \times \mathrm{Y_p} \times \mathrm{t} \times \mathrm{A} \times 10^{-4}}{\mathrm{Q}}$$

Where n is the number of electrons transferred, F is the faraday constant (96485 C mol^{-1}), Y_p is the products formation rate (μmol cm^{-2} h^{-1}), t is time (h) for NORR, A is the electrode area (cm^2), and Q is the quantity of charge consumed during the reaction (C).

The Energy Efficiency (EE) can be calculated using the following formula:

$$EE = \frac{E_0 \times FE}{E_c}$$

Where E_o is the theoretical thermodynamic cell voltage (V), and E_c is the applied cell voltage (V).

The STF can be calculated using the following formula:

$$STF = \frac{P_{EC-out}}{P_{solar-in}} = \frac{E_0 \times J_{solar} \times FE}{P_{solar}}$$

Where E_o is the theoretical thermodynamic cell voltage (V), J_{solar} is the solar current density (mA cm^{-2}), and P_{solar} is the solar energy input (mW cm^{-2}).

The turnover frequency (TOF) was calculated as follows:

$$TOF = \frac{j \times M_w}{n \times F \times \mathrm{r}}$$

Where i is the current density (mA cm^{-2}), M_w is the molecular weight of nickel (g mol^{-1}), r is the catalyst loading (mg cm^{-2}), and F is the faraday constant (96485 A s mol^{-1}).

Note S1. Determination TOF at an overpotential of 550 mV for all Ni@NC catalysts

The amount of Ni loading in the catalyst is measured using ICP-OES (Table S4). The following equation is used to calculate the TOF value,

$$TOF(s^{-1}) = \frac{\boldsymbol{j\ (mA\ cm^{-2}) \times M_W\ (g\ mol^{-1})}}{\boldsymbol{n \times F(A\ s\ mol^{-1}) \times \mathrm{r}\ (mg\ cm^{-2})}}$$

The NORR current density at the overpotential (ŋ) of 550 mV was taken for all the catalysts,

$TOF_{(ŋ=550\ mV)}$ = [(3.77×10^{-3}) (58.69)] / [(5) (96485) (0.551×10^{-3})]

= 0.000832 s^{-1}

Ni@NC-1 = 0.000832 s^{-1}

Similarly, $TOF_{(ŋ=550\ mV)}$ was calculated for the remaining catalysts as,

Ni@NC-2 = 0.000874 s^{-1}

Ni@NC-3 = 0.00104 s^{-1}

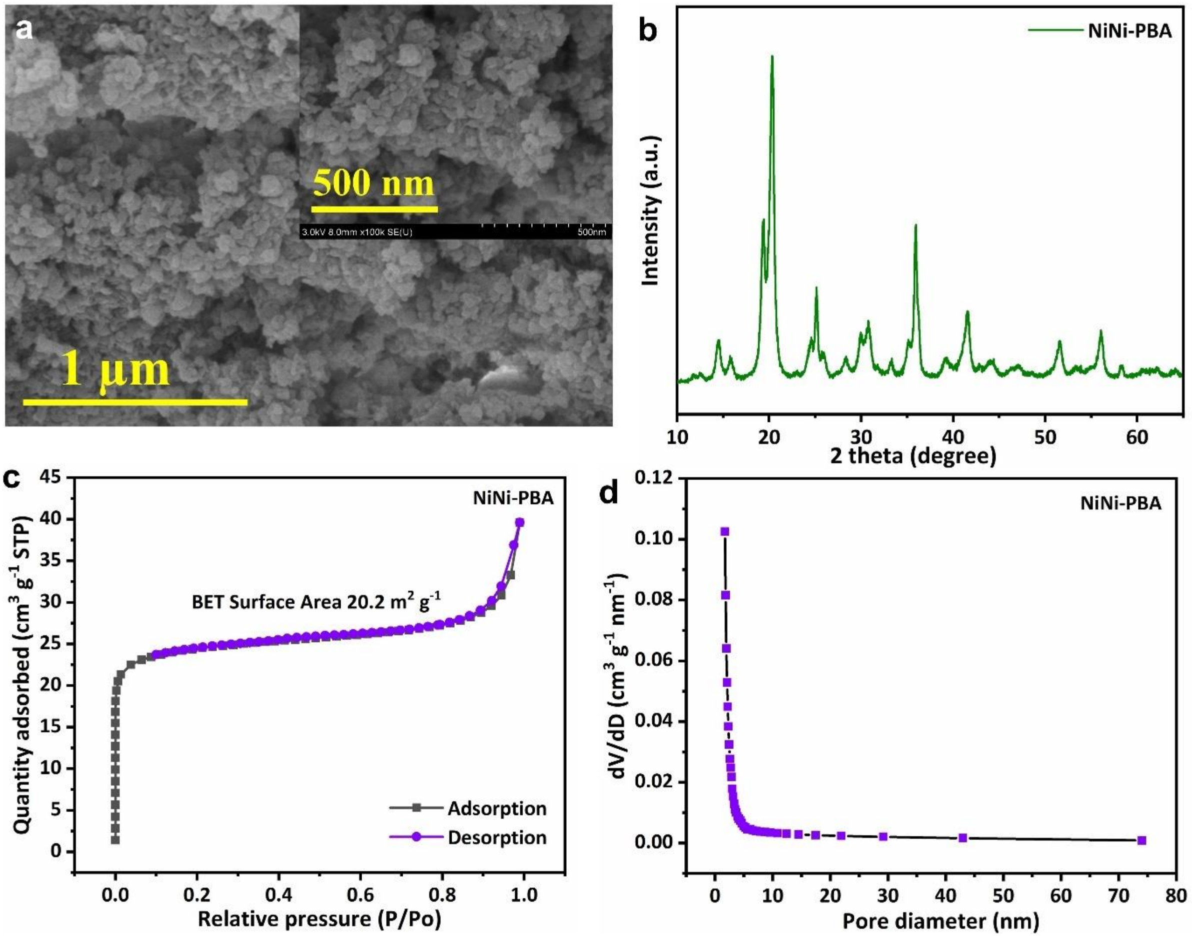

Figure S1. a) FESEM, inset) high magnification FESEM image, b) XRD, c) N_2 adsorption-desorption isotherms, and d) pore-size distribution of NiNi-PBA.

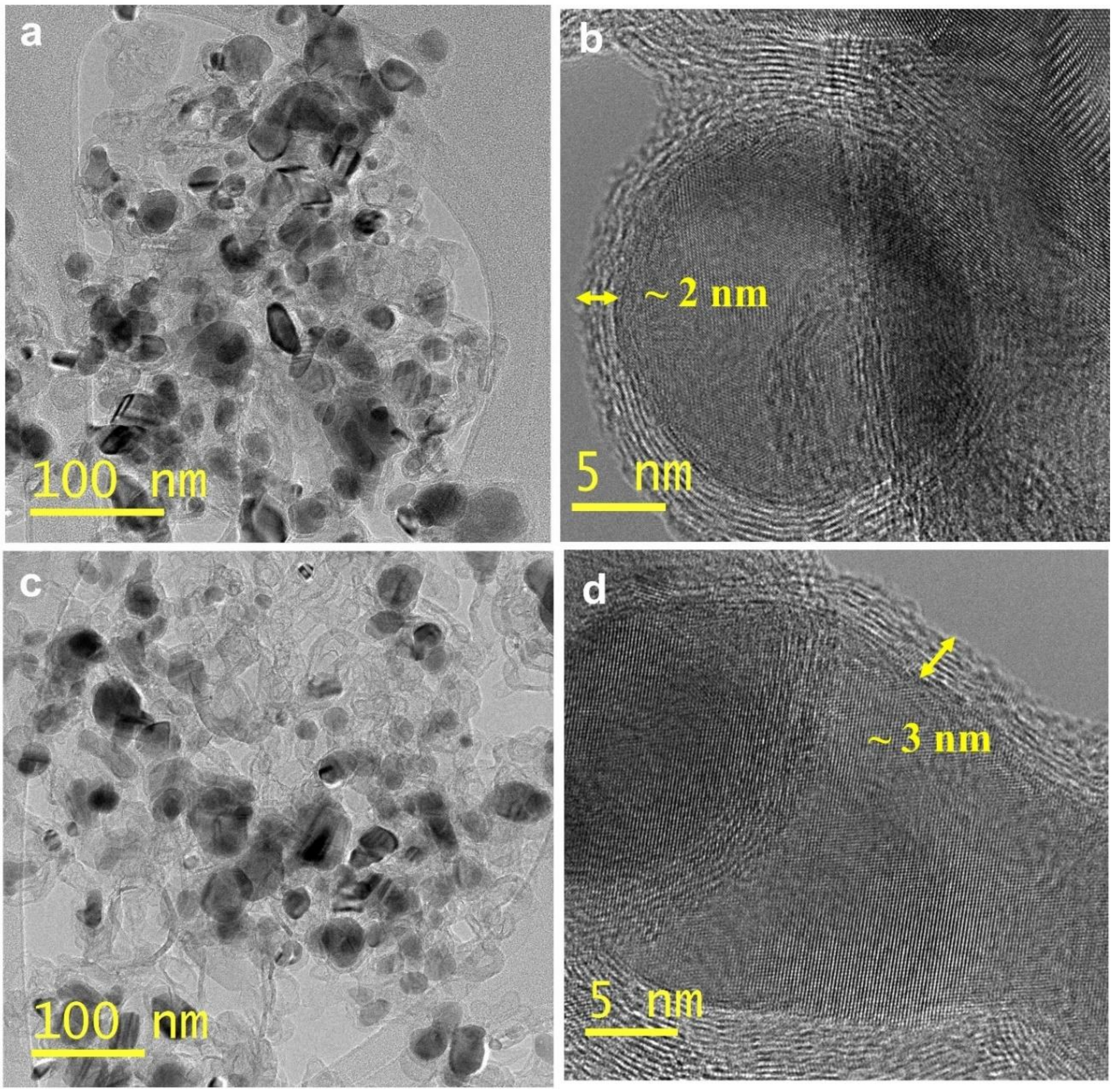

Figure S2. TEM and HRTEM images of the a-b) Ni@NC-1 and c-d) Ni@NC-2.

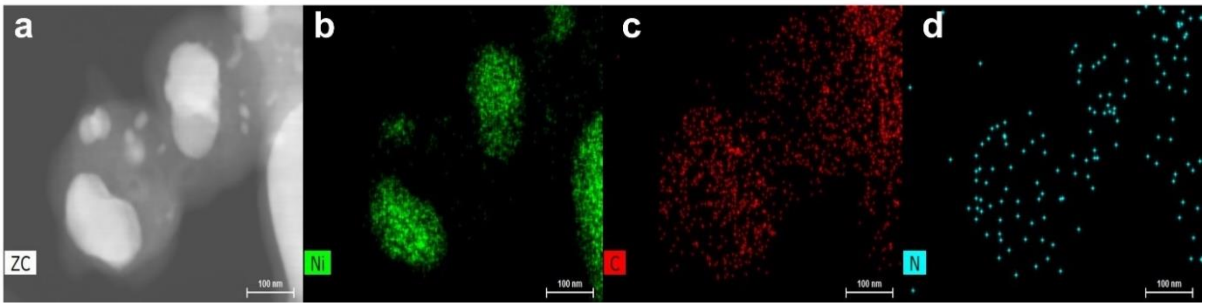

Figure S3. a) STEM image of the core-shell Ni@NC-3, TEM-EDS mapping images of b) Nickel, c) Carbon, and d) Nitrogen.

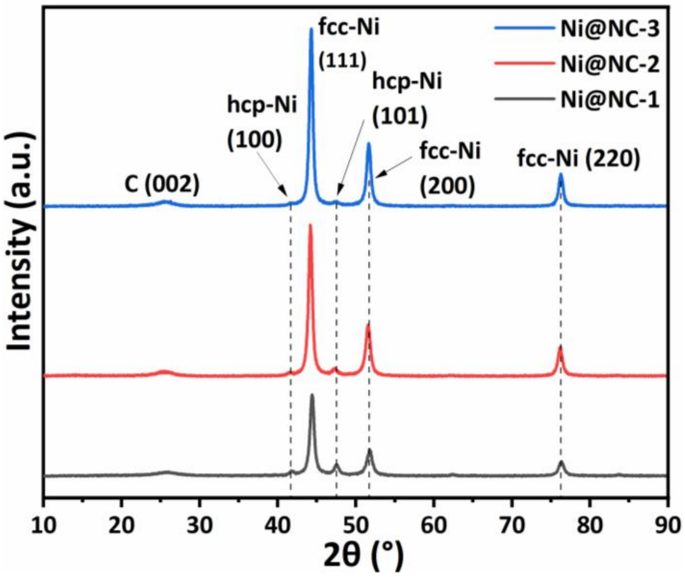

Figure S4. XRD patterns of the core-shell Ni@NC catalysts obtained at different temperatures from 800 to 600 °C.

Figure S4 shows that the core-shell Ni@NC catalysts possess two different crystal structures: face centered cubic (FCC) and hexagonal close packing (HCP) of Ni nanoparticles. FCC crystal structure is a stable phase of Ni, while HCP is a metastable phase usually formed at low calcination temperature. The peaks at 44.4°, 51.5°, and 76.4° correspond to (111), (200), and (220) facets of FCC nickel. On the other hand, the peaks at 41.9° and 47.7° correspond to the HCP Ni (100) and (101) planes, respectively. The weak peak appeared at 26.4° ascribed to the (002) plane of graphitic carbon. The results imply that when temperature increased from 600 to 800 °C, the crystallinity of the nanostructures increased with the diminishing intensity of HCP nickel peaks. Thus, Ni@NC-3 contains predominantly FCC-phase Ni nanoparticles surrounded by a multilayer of carbon framework compared to other control samples.

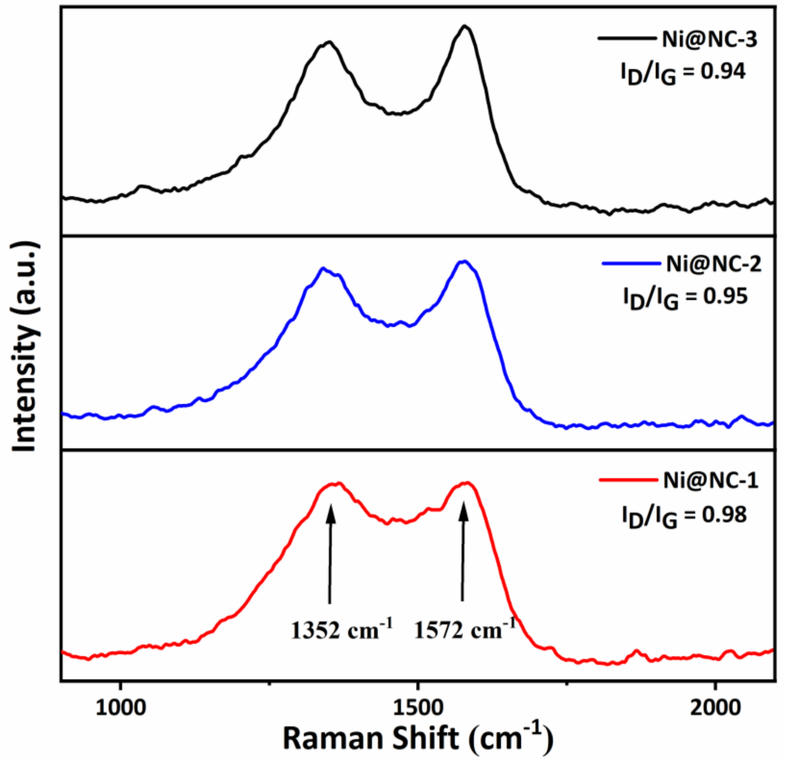

Figure S5. Raman spectra of the Ni@NC catalysts

Two typical peaks at 1350 cm^{-1} (D-band) and 1580 cm^{-1} (G-band) were observed for Ni@NC catalysts, which are related to the defective and ordered carbon domains, respectively. The intensity ratios of D- and G-band (I_D/I_G) of the samples decreased with the increase in annealing temperature. It indicates that the graphitization of the catalysts increases with an increase in annealing temperature. Among the catalysts, Ni@NC-3 shows the lowest I_D/I_G ratio of 0.94, implying the presence of ordered carbon layers with a high graphitization degree.

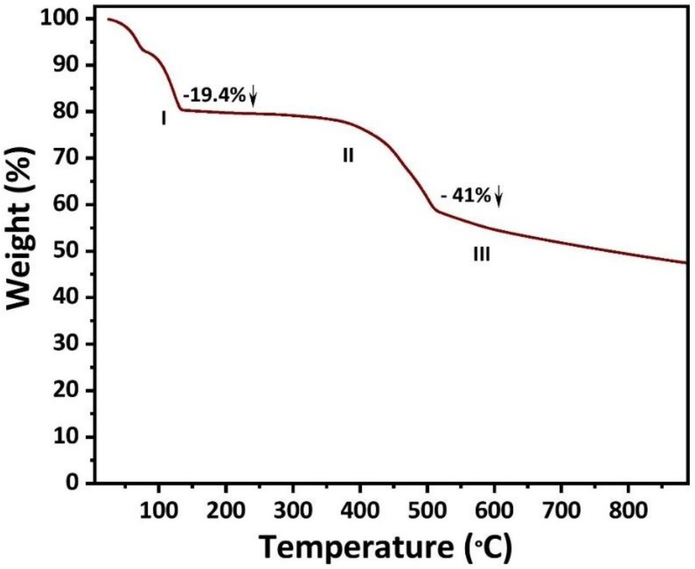

Figure S6. TGA curve of the NiNi-PBA under N_2 atmosphere.

The three distinct decomposition steps: I (27–131 °C), II (131–510 °C), and III (510–1000 °C). The water molecules associated with the analog structure were removed in the first step. It is followed by the destruction of ligand pairs and the removal of cyanide groups from the PB structure in the second step. After 510 °C, the complex is stable, and only a slight decrease in the weight corresponds to the removal of nitrogen content which is already confirmed by the CHNS results.

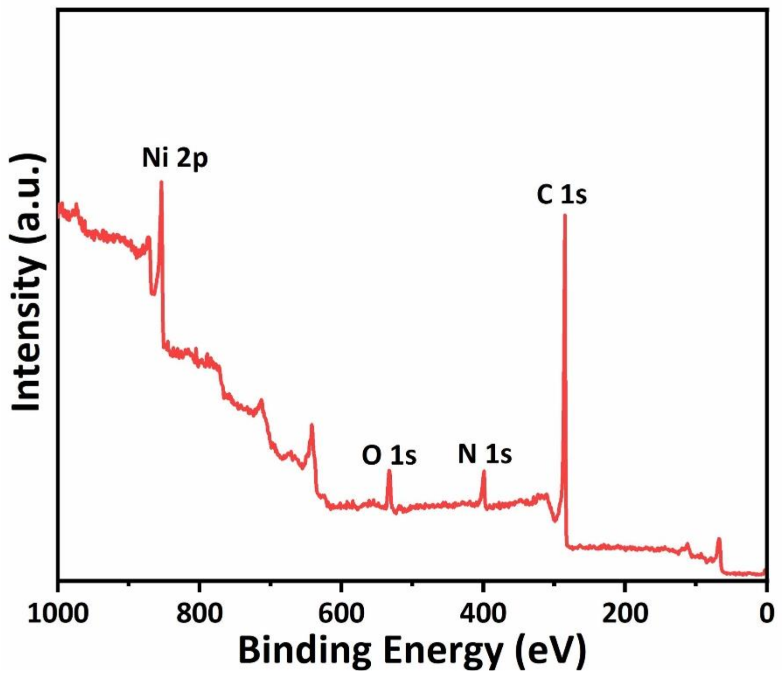

Figure S7. XPS survey spectrum of the Ni@NC-3 catalyst.

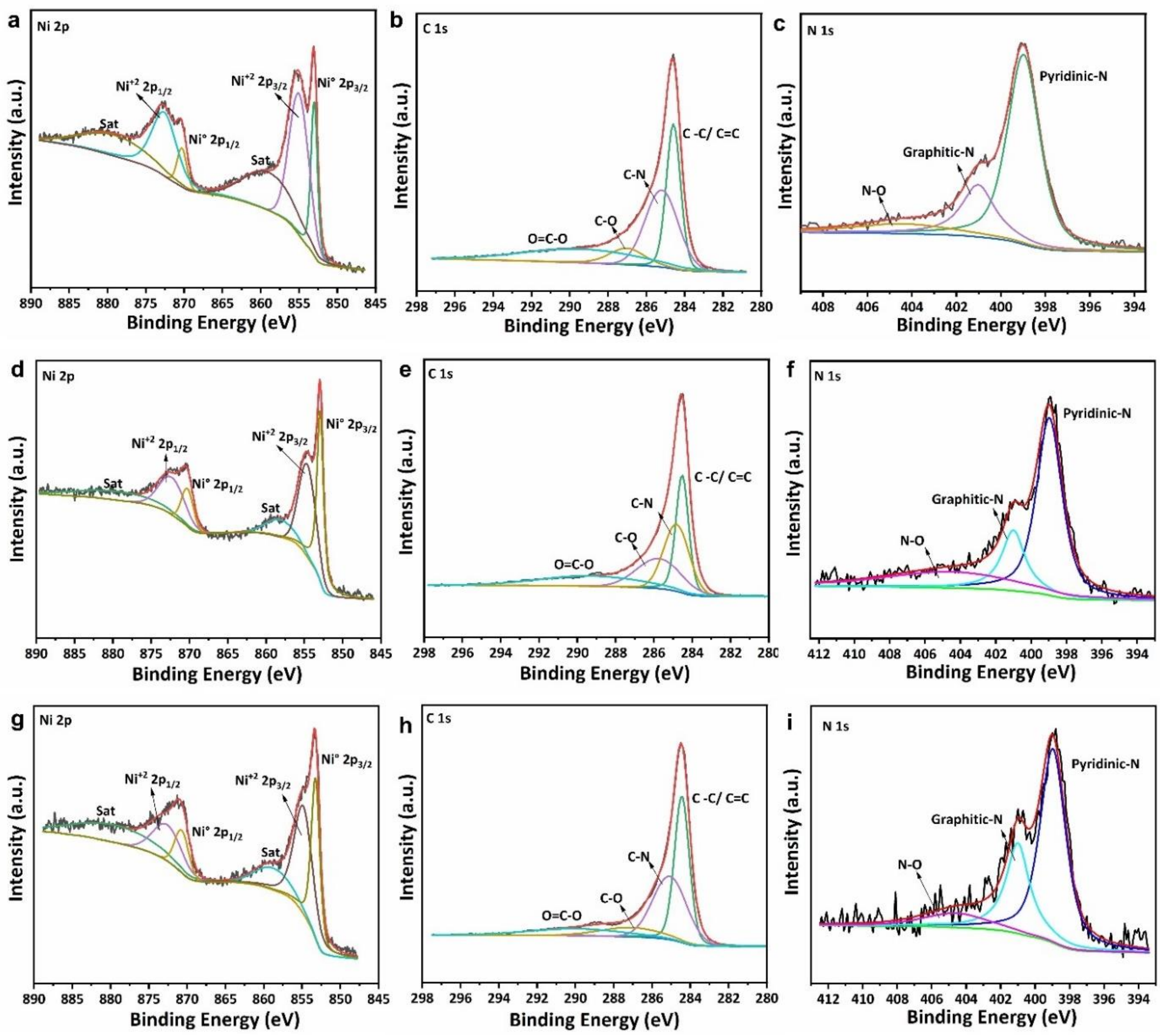

Figure S8. High-resolution XPS spectra of the core-shell Ni@NC catalysts. Ni@NC-1: a) Nickel (Ni 2p), b) Carbon (C 1s), and c) Nitrogen (N 1s). Ni@NC-2: d) Nickel (Ni 2p), e) Carbon (C 1s), and f) Nitrogen (N 1s). Ni@NC-3: g) Nickel (Ni 2p), h) Carbon (C 1s), and i) Nitrogen (N 1s).

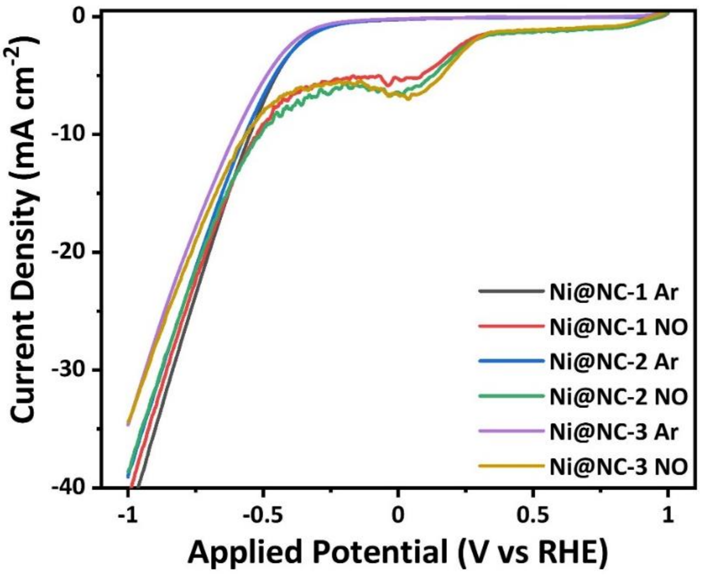

Figure S9. Linear sweep voltammetry study of all the core-shell Ni@NC catalysts in 0.1 M HCl at a scan rate of 5 mV s^{-1}.

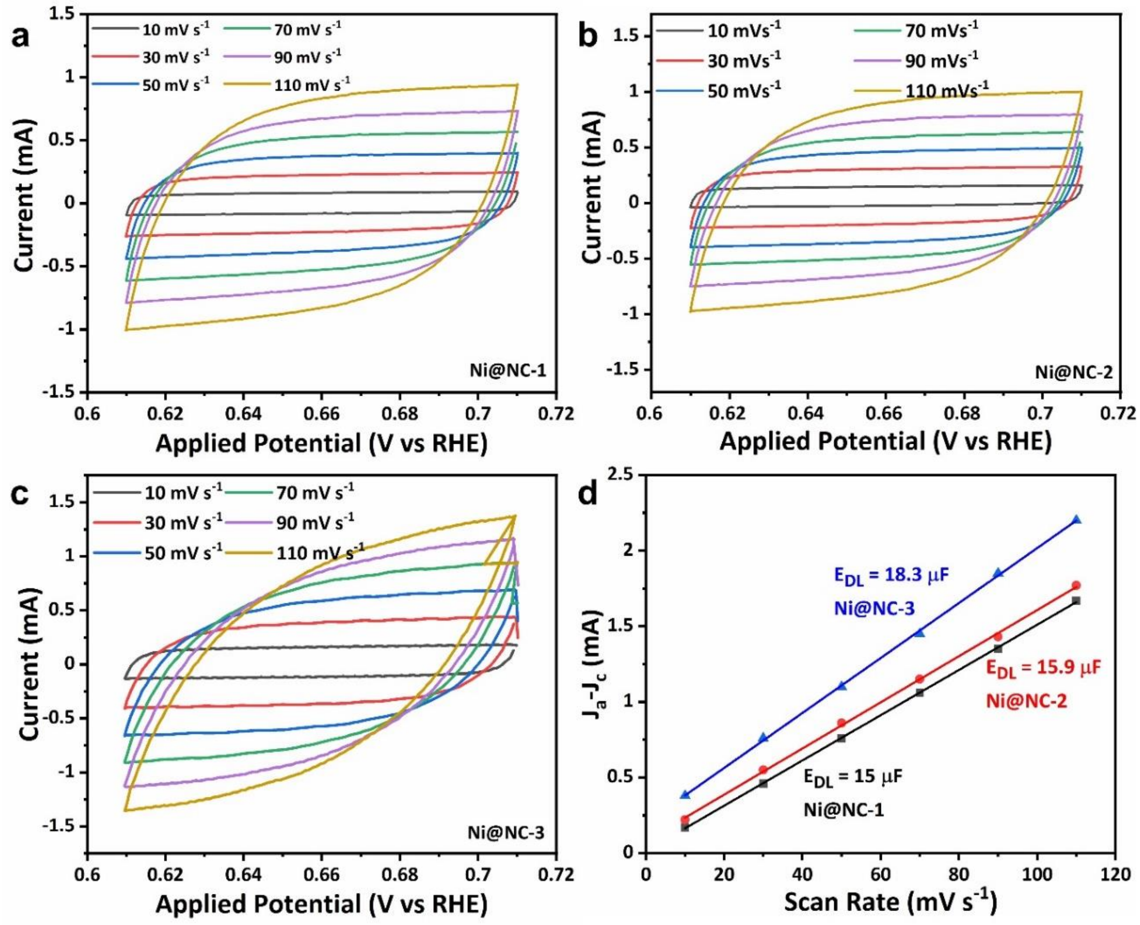

Figure S10. Electrochemical active surface area calculation of a) Ni@NC-1, b) Ni@NC-2, and c) Ni@NC-3. d) Comparison of E_{DL} value of all the Ni@NC catalysts. The ECSA of all the Ni@NC catalysts was calculated in the Ar medium in the potential range of 0.61 to 0.71 $V_{RHE.}$

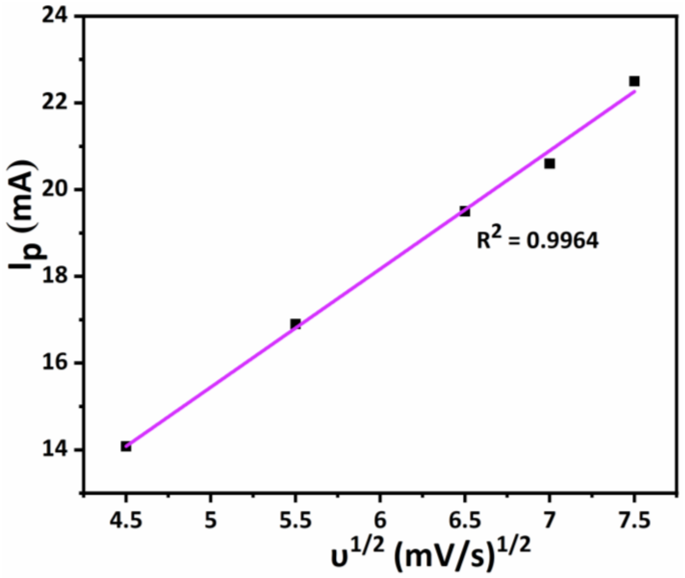

Figure S11. The plot of peak current (I_p)and the square root of scan rate (v) obtained from the polarization curves measured at different scan rates (20, 30, 40, 50, and 60 mV s^{-1}) in NO saturated electrolyte.

Randles-Sevcik equation

$$I_p = 2.69\ x\ 10^5\ A\ C\ n^{3/2}\ D^{1/2} \upsilon^{1/2}$$

where,

I_{peak} is peak current (A)

n is number of electrons transferred

F is Faraday constant (96485 C mol^{-1})

D is the diffusivity of NO species (2.5x10^{-5} cm^2 s^{-1})

A is the active electrode area (cm^2)

C is Bulk NO concentration (1.4 x 10^{-3} mol cm^{-3})

υ is scan rate (V s^{-1})

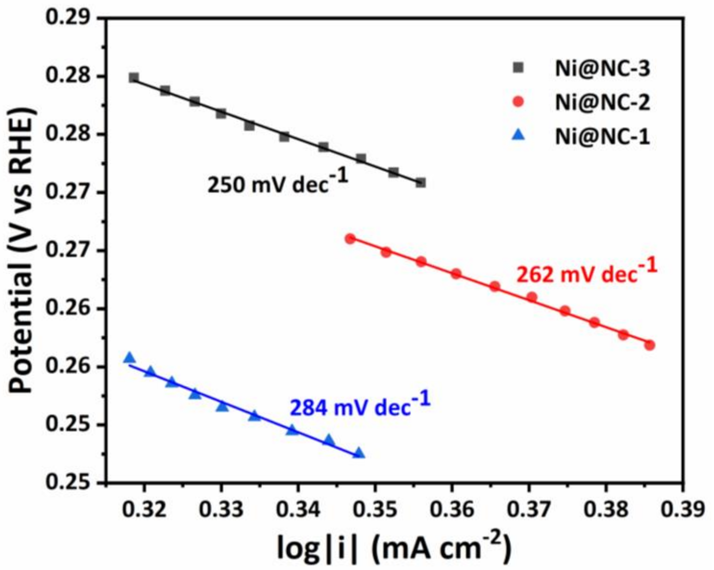

Figure S12. Tafel slopes obtained from the polarization curves for all the Ni@NC catalysts

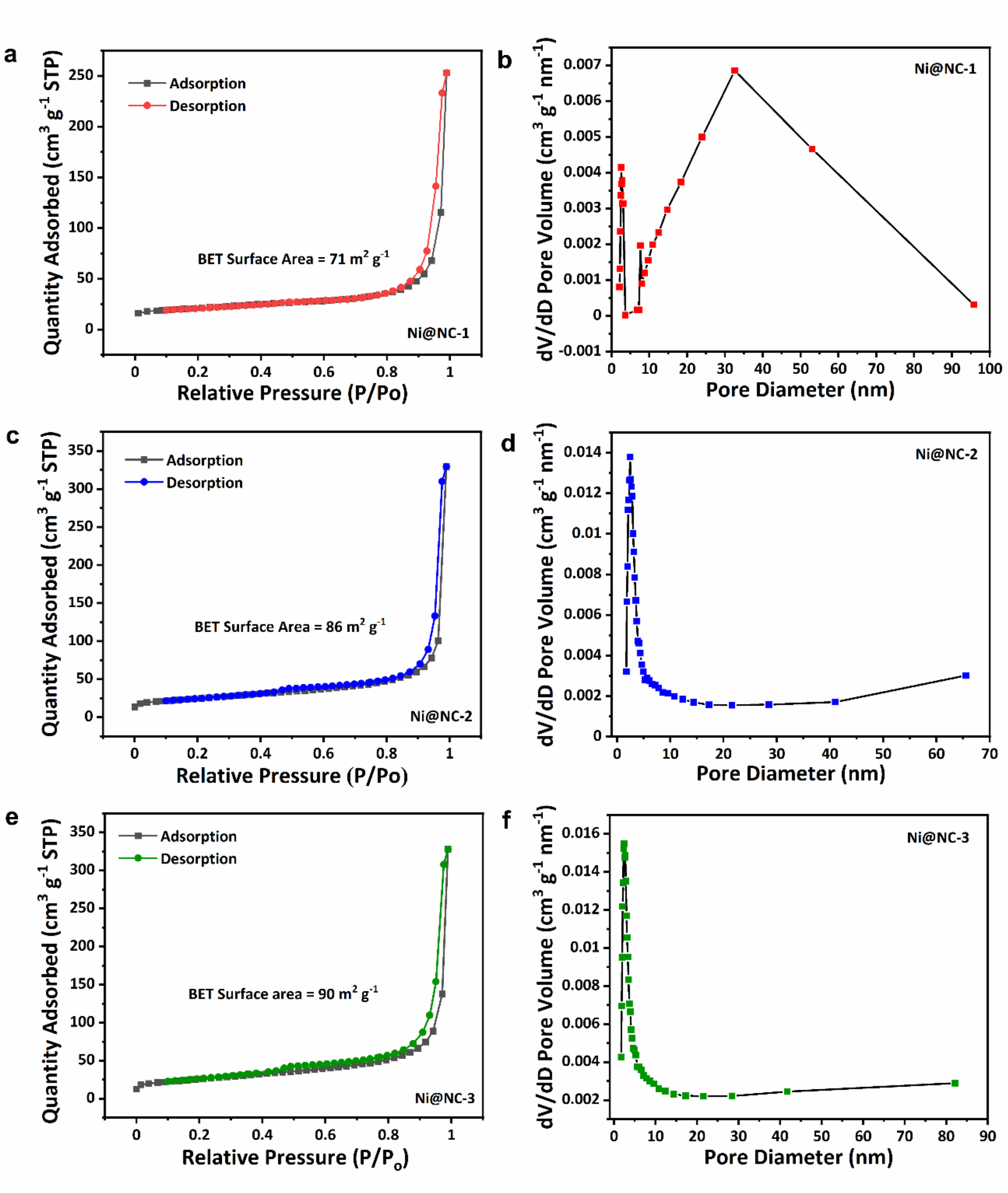

Figure S13. N_2 adsorption-desorption isotherms and pore-size distribution of Ni@NC catalysts.

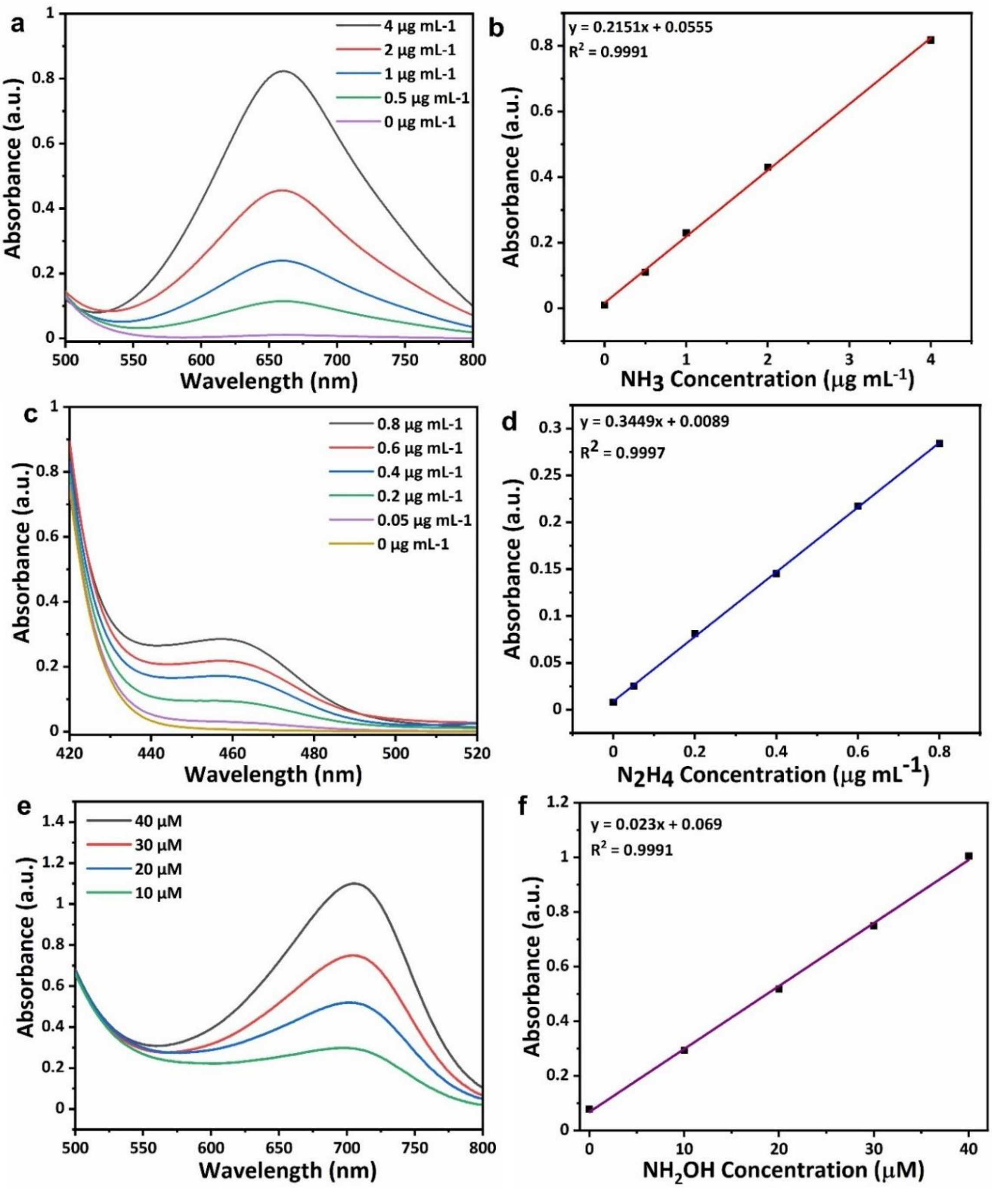

Figure S14. UV-vis calibrations of NH_3, N_2H_4, and NH_2OH. a, b) UV-vis spectra and calibration curve of standard NH_3 solution, c, d) UV-vis spectra and calibration curve of standard N_2H_4 solution, e, f) UV-vis spectra and a calibration curve of standard NH_2OH solution

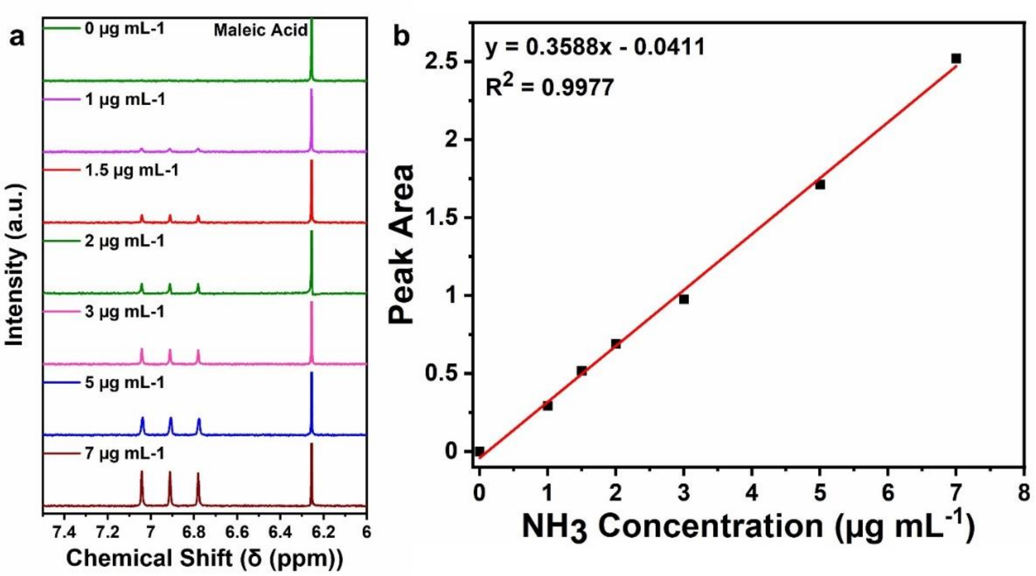

Figure S15. ¹H NMR quantification of NH_3 (a) ¹H NMR spectra of various concentrations of standard NH_3 containing 0.1 м HCl, (b) calibration curve used to measure NH_3 concentration.

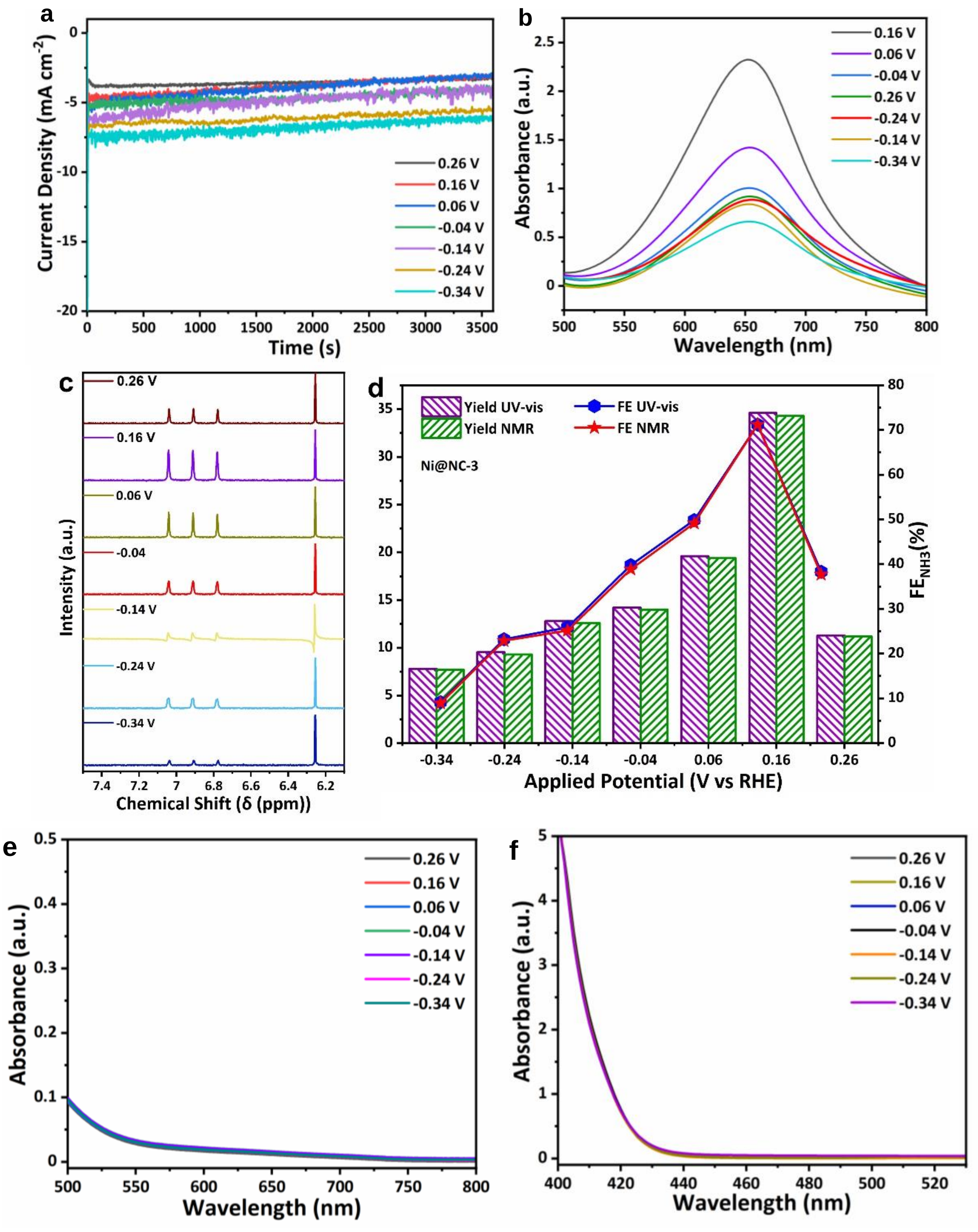

Figure S16. Electrocatalytic NORR performance of the Core-shell Ni@NC-3. a) CA curves at the various potential in 0.1 M HCl, b) UV-vis absorption spectra at different potentials after 1 h electrolysis for NH_3 quantification, c) ^{1}H NMR of the same electrolysis for NH_3 quantification, d) corresponding NH_3 yield rate and FE_{NH3} by UV-vis and ^{1}H NMR quantification, e) UV-vis absorption spectra at different potentials after 1 h electrolysis for NH_2OH quantification, f) UV-vis absorption spectra at different potentials after 1 h electrolysis for N_2H_4 quantification

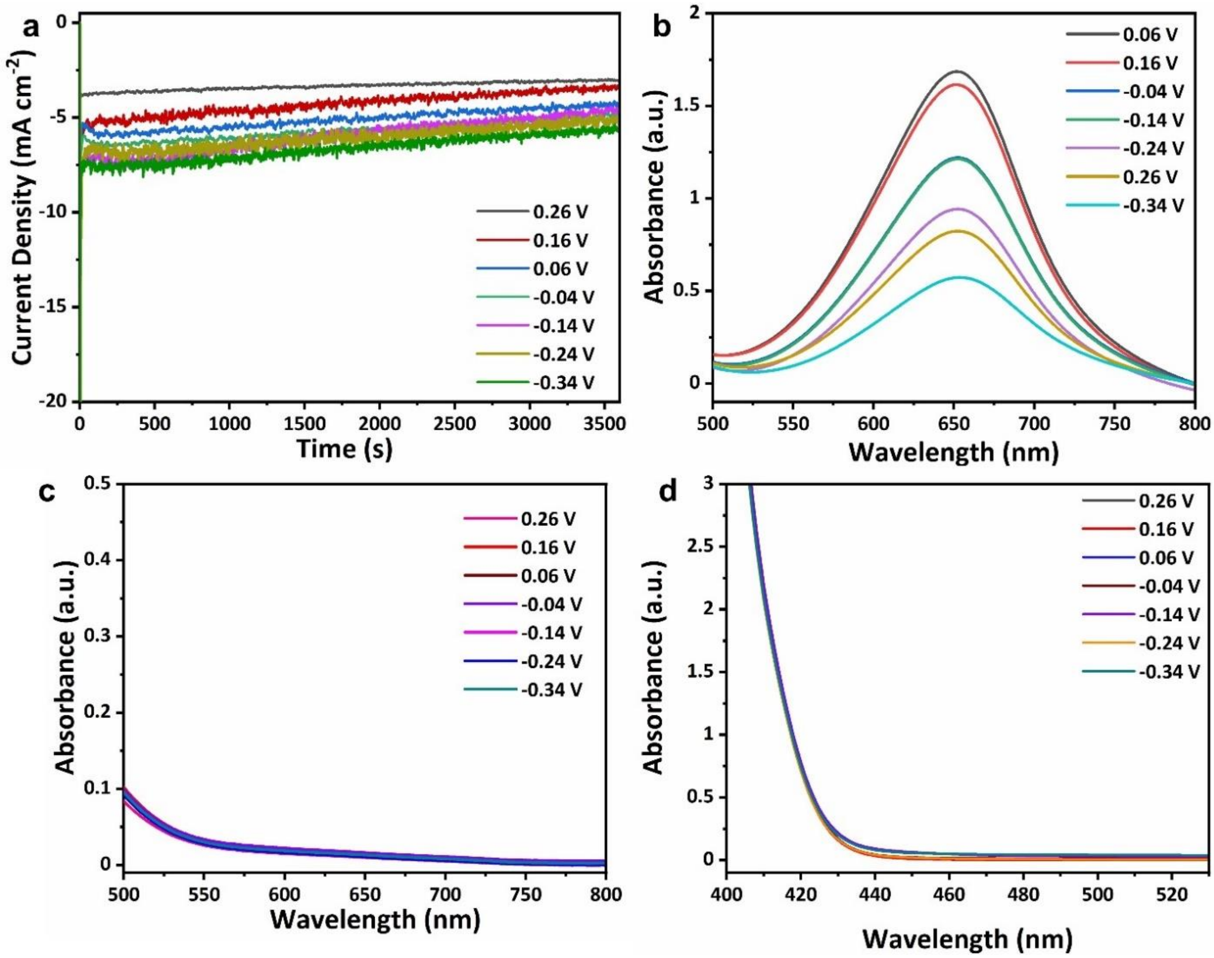

Figure S17. Electrocatalytic NORR performance of the Core-shell Ni@NC-1. a) chronoamperometry curves of the catalyst coated on carbon cloth substrate at the various potential in 0.1 M HCl, b) UV-vis absorption spectra at different potentials after 1 h electrolysis for NH_3 quantification, c) UV-vis absorption spectra at different potentials after 1 h electrolysis for NH_2OH quantification, d) UV-vis absorption spectra at different potentials after 1 h electrolysis for N_2H_4 quantification

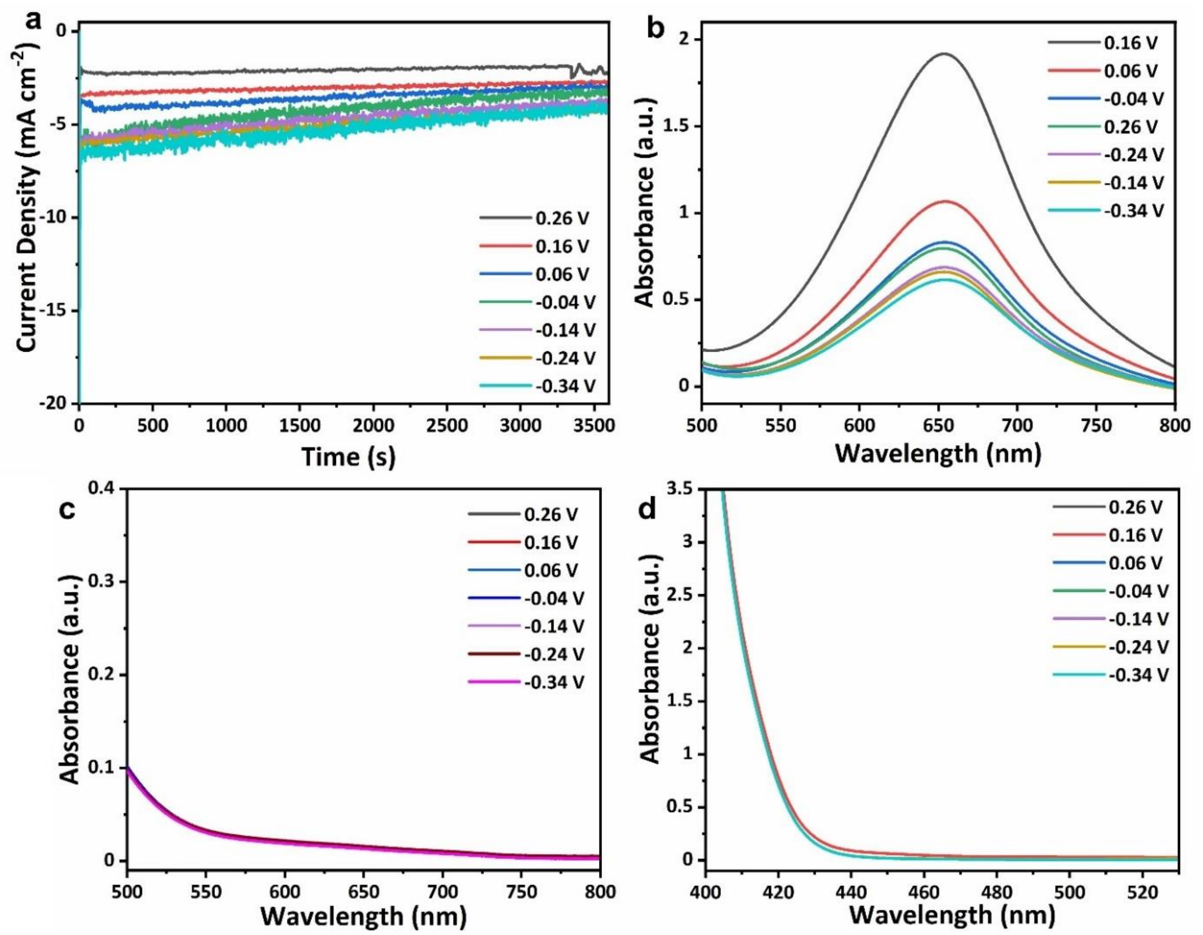

Figure S18. Electrocatalytic NORR performance of the Core-shell Ni@NC-2. a) chronoamperometry curves of the catalyst coated on carbon cloth substrate at the various potential in 0.1 M HCl, b) UV-vis absorption spectra at different potentials after 1 h electrolysis for NH_3 quantification, c) UV-vis absorption spectra at different potentials after 1 h electrolysis for NH_2OH quantification, d) UV-vis absorption spectra at different potentials after 1 h electrolysis for N_2H_4 quantification

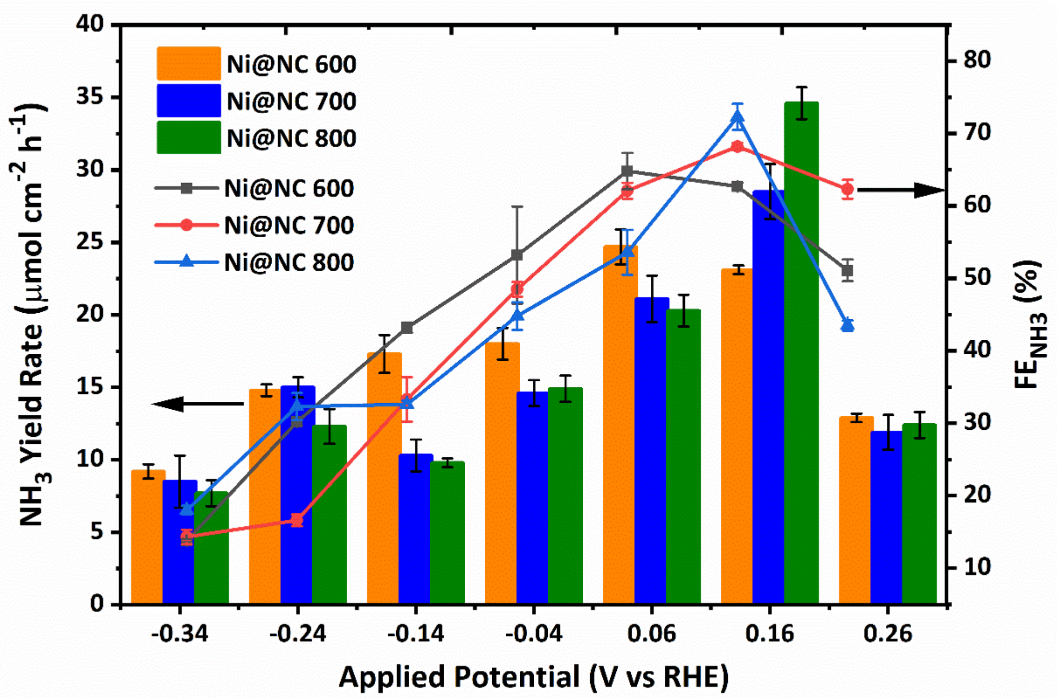

Figure S19. Comparison of electrocatalytic NORR performance of all the Core-shell Ni@NC catalysts in terms of NH_3 yield and FE_{NH3}. Error bars indicate the standard error from the three repeated measurements.

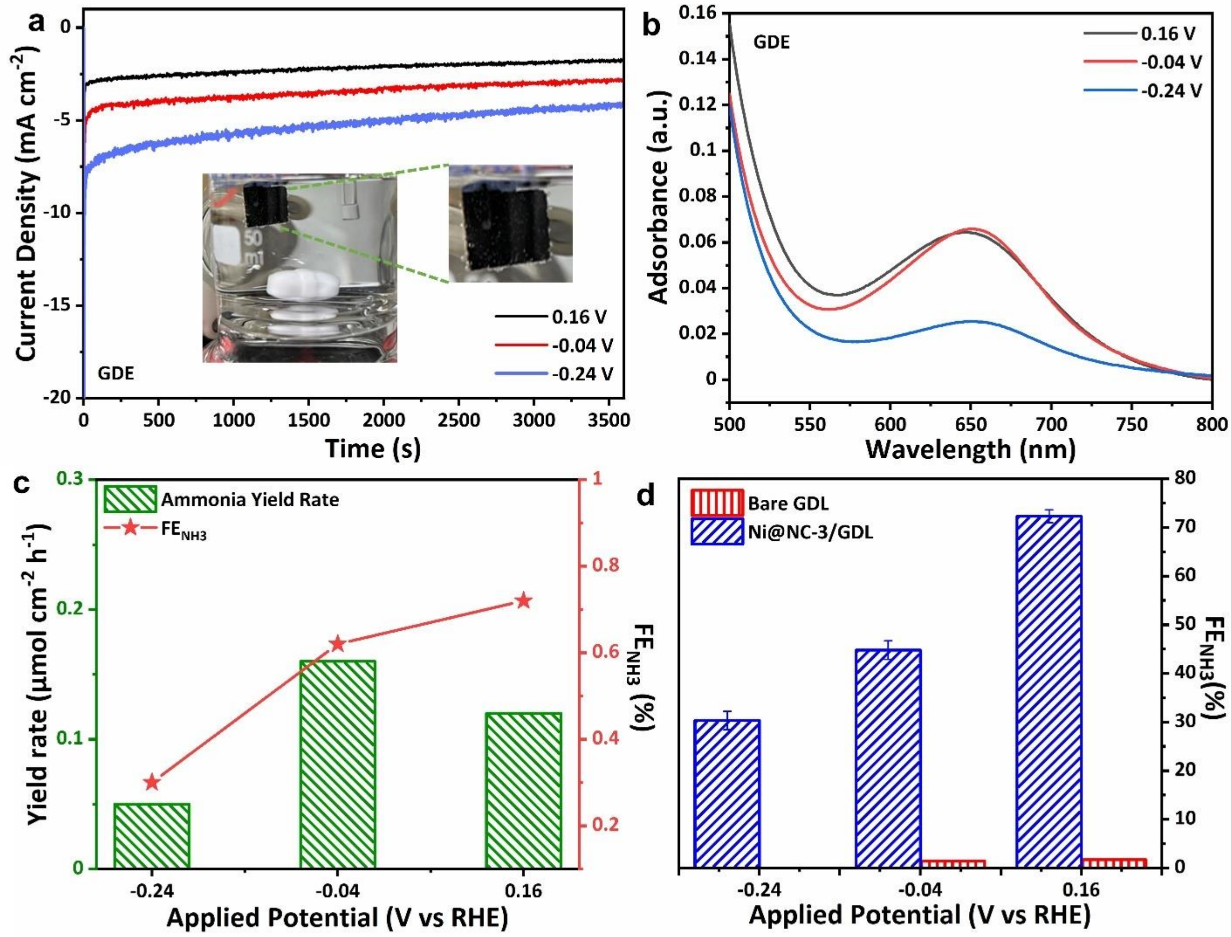

Figure S20. Electrocatalytic NORR performance of bare GDE. a) CA at different potentials (inset: gas products evolved during the CA study), b) corresponding UV-vis absorbance of the analyte stained with indophenol indicator, c) corresponding NH_3 yield and FE_{NH3}, and d) comparison of NH_3 yield and FE_{NH3} of bare GDE and Ni@NC-3 catalyst.

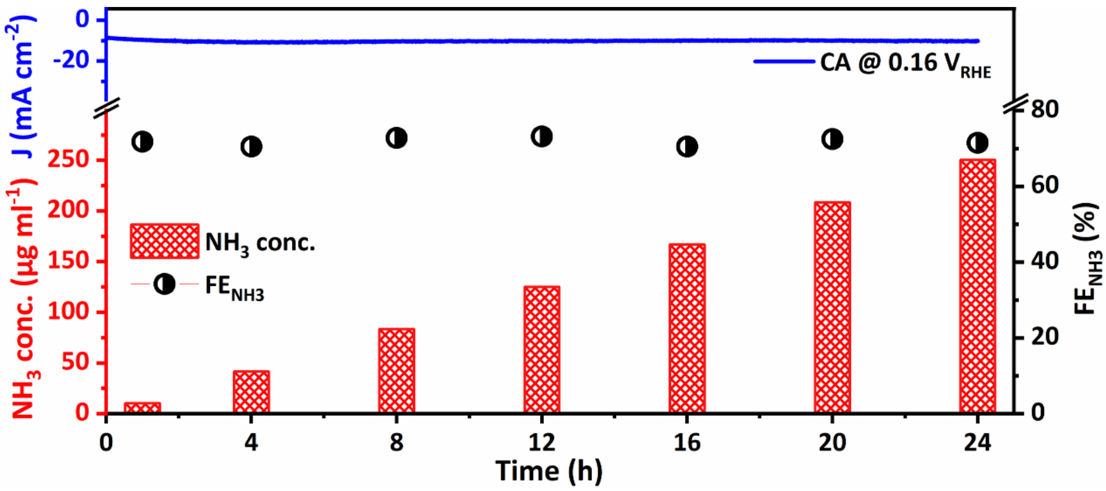

Figure S21. Long-term stability test for continuous generation of NH_3 on Ni@NC-3 at an applied potential of 0.16 V_{RHE}.

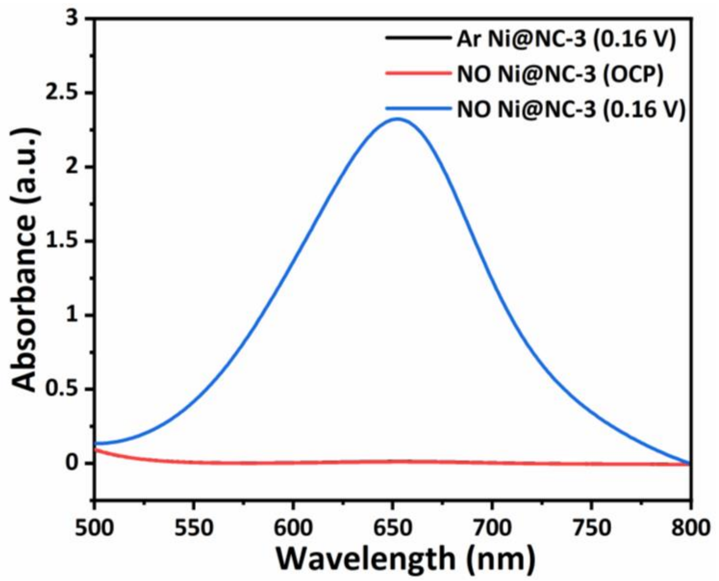

Figure S22. UV–vis spectra of the analyte under different control experiments.

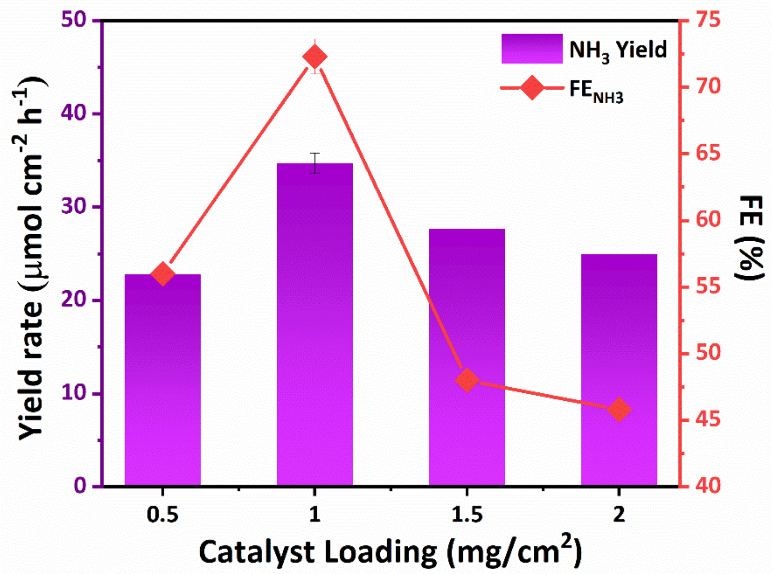

Figure S23. NH_3 yield rate and FE_{NH3} of Ni@NC-3 with different catalyst loading at 0.16 V_{RHE}.

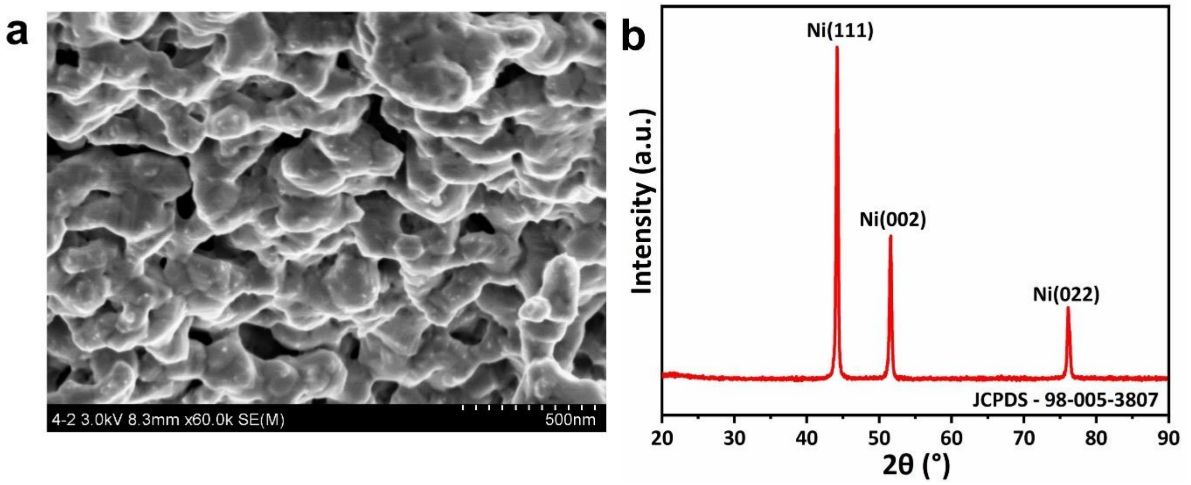

Figure S24. a) FESEM and b) XRD pattern of Ni nanoparticles.

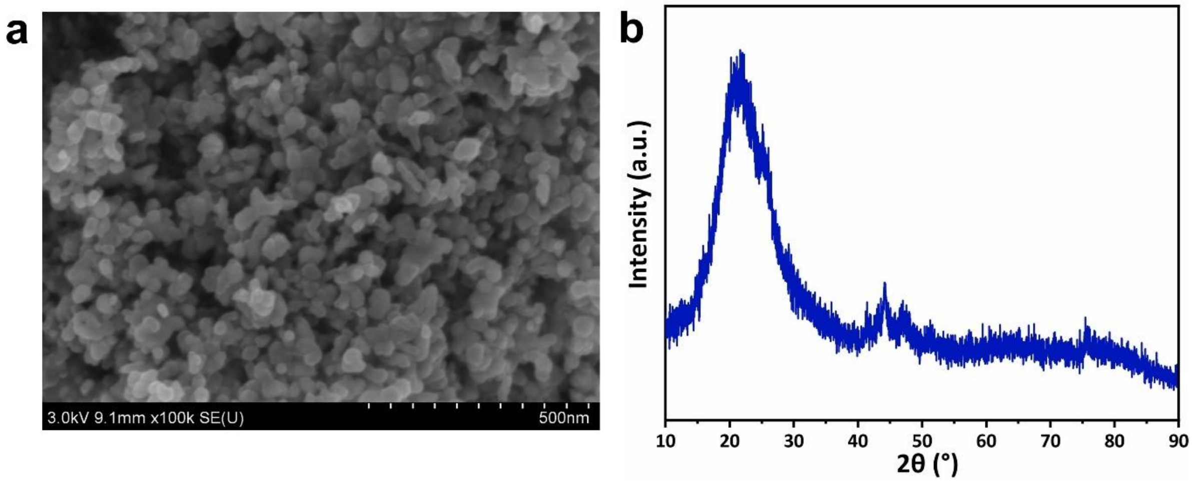

Figure S25. a) FESEM and b) XRD pattern of NC.

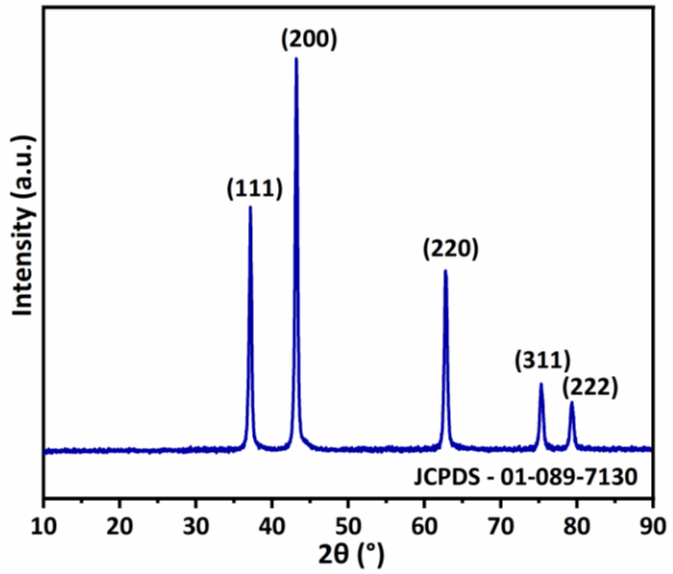

Figure S26. XRD pattern of NiO nanoparticle.

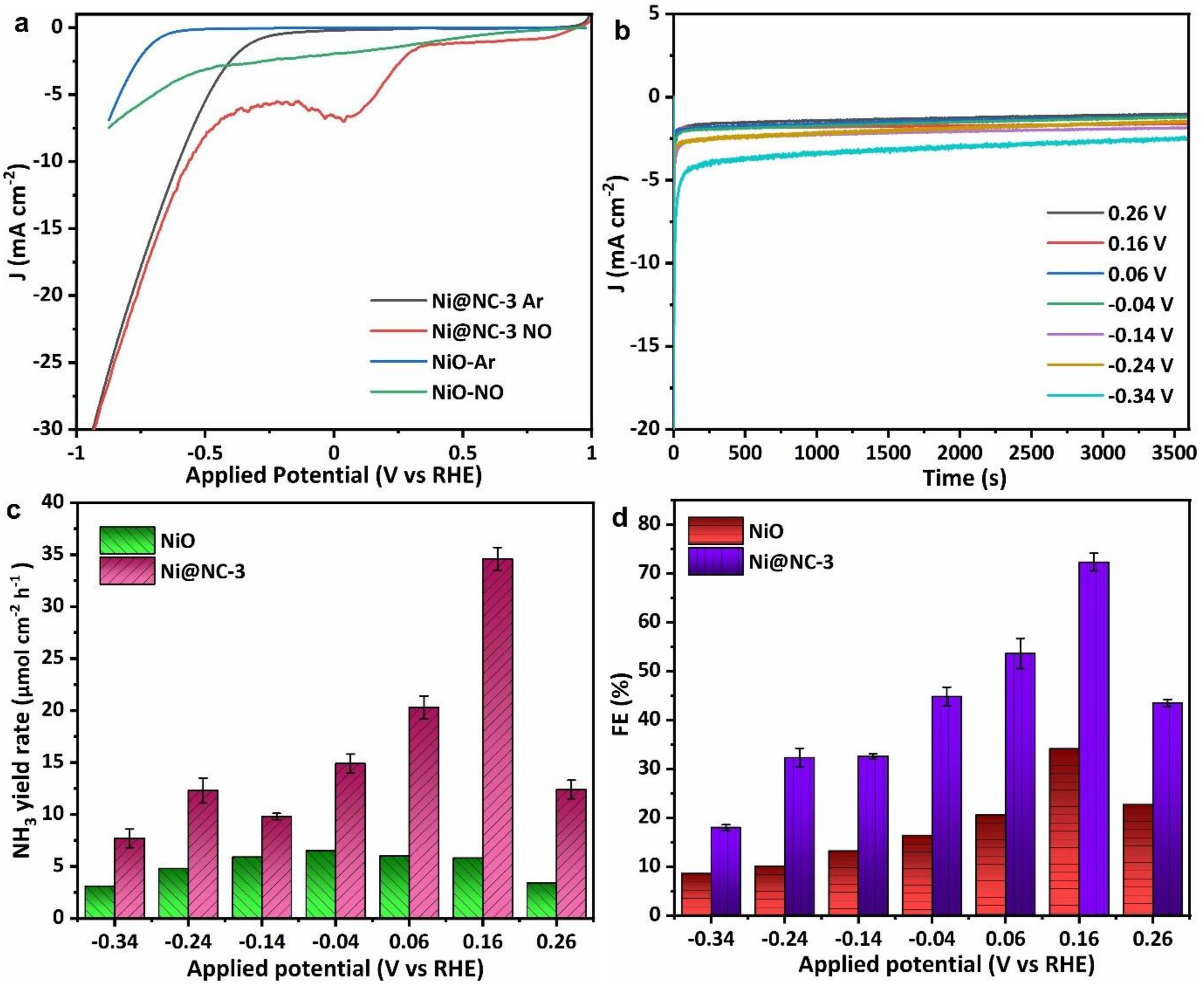

Figure S27. Electrochemical NORR performance of NiO catalyst in 0.1 M HCl. a) Polarization curves obtained on Ni@NC-3 and NiO in Ar and NO-saturated medium. b) Chronoamperometry curves obtained at various potential in 0.1 M HCl. c) Comparison of NH_3 yield rate obtained for NiO and Ni@NC-3 catalysts at various potentials and d) the corresponding FE_{NH3} of NiO and Ni@NC-3 catalysts at various potentials.

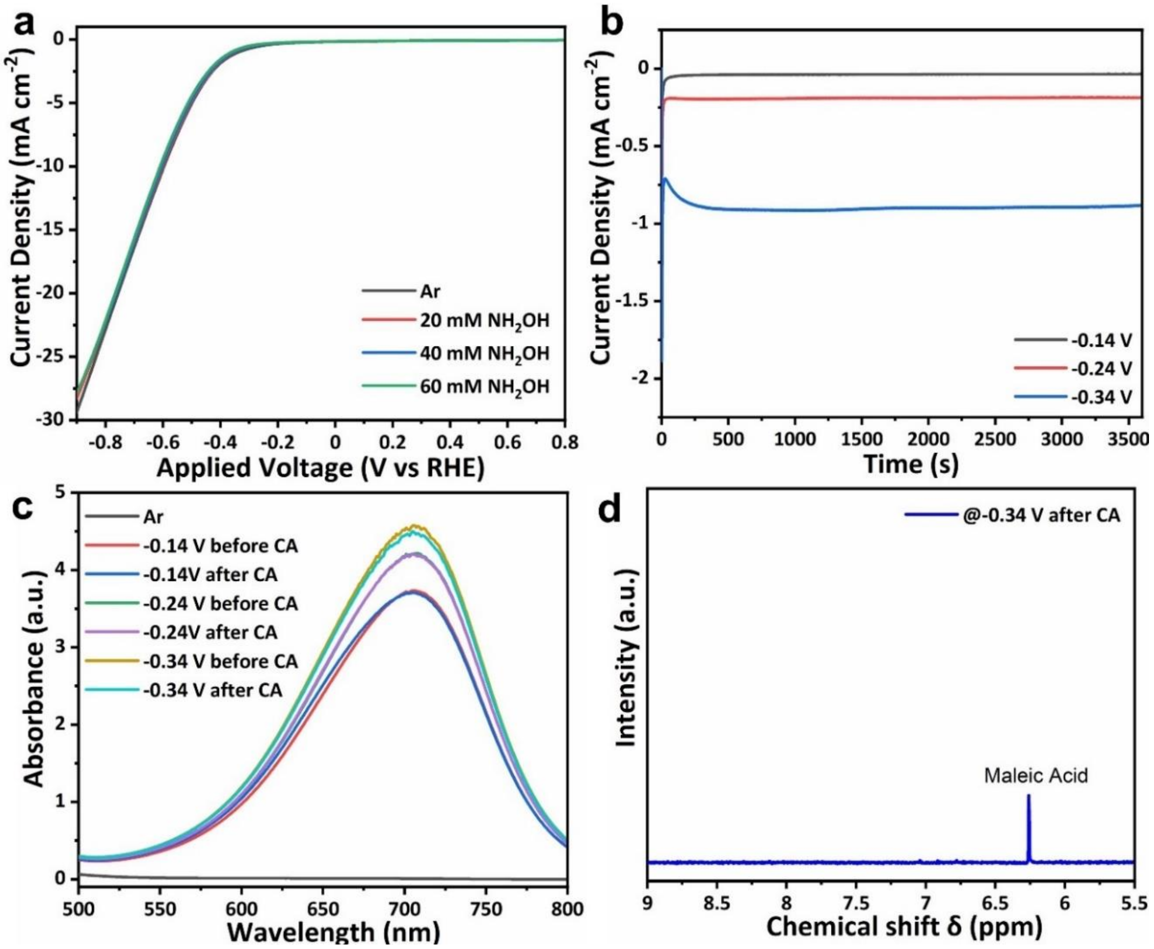

Figure S28. Electrocatalytic NH_2OH reduction on Ni@NC-3**.** a) polarization curves with 20 mM, 40 mM, and 60 mM of NH_2OH and without NH_2OH under Ar-medium. b) CA profile depending on the applied potential vs RHE in 20 mM NH_2OH in 0.1 M HCl. c) corresponding UV-vis spectra before and after the CA study to quantify NH_2OH. d) ^{1}H NMR of the electrolyte taken after the CA at -0.34V to detect NH_3.

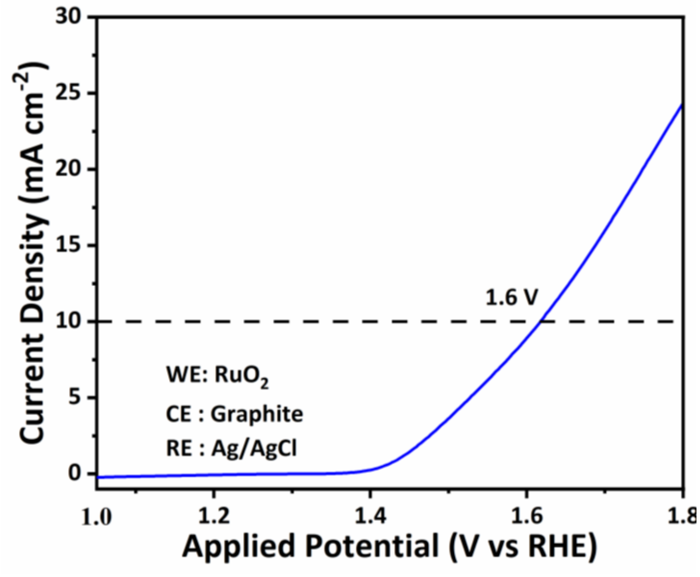

Figure S29. Polarization curve of RuO_2 for OER electrocatalysis in 0.1 м HCl.

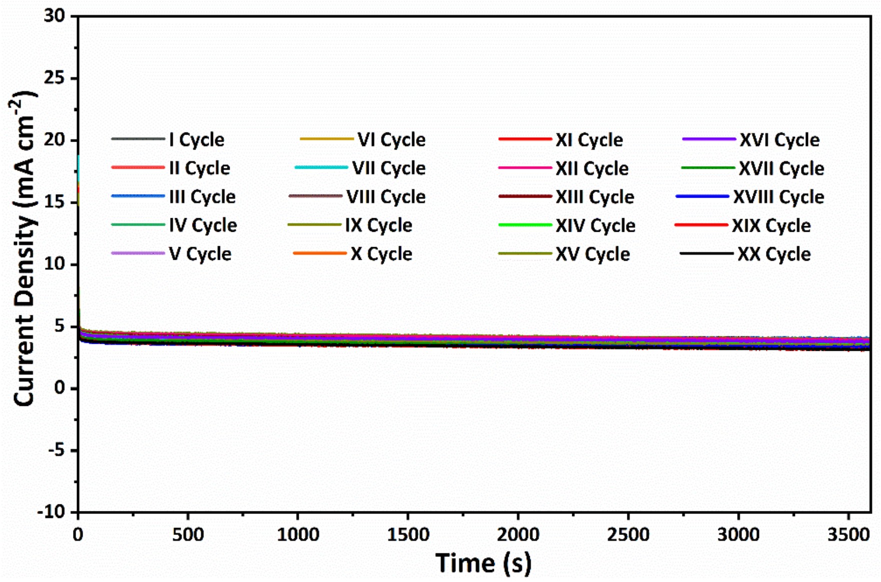

Figure S30. CA curves of the repeated cycling stability of full-cell NORR-OER electrolyzer for 20 cycles.

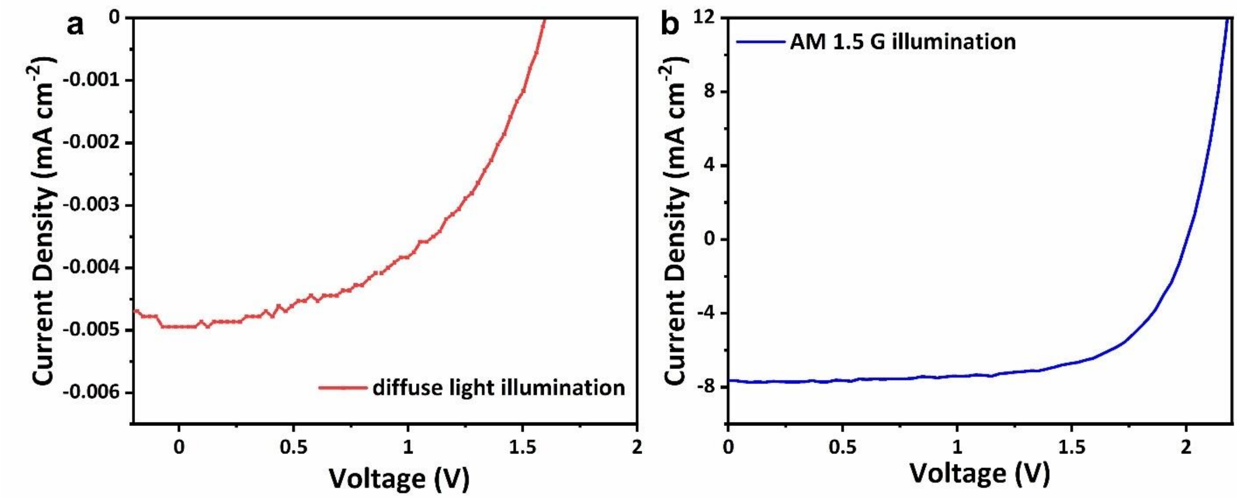

Figure S31. : Solar cell characterization. a) j-V characteristics of the solar cell under ambient diffuse light illumination. b) j-V characteristics of the solar cell under AM 1.5 G illumination.

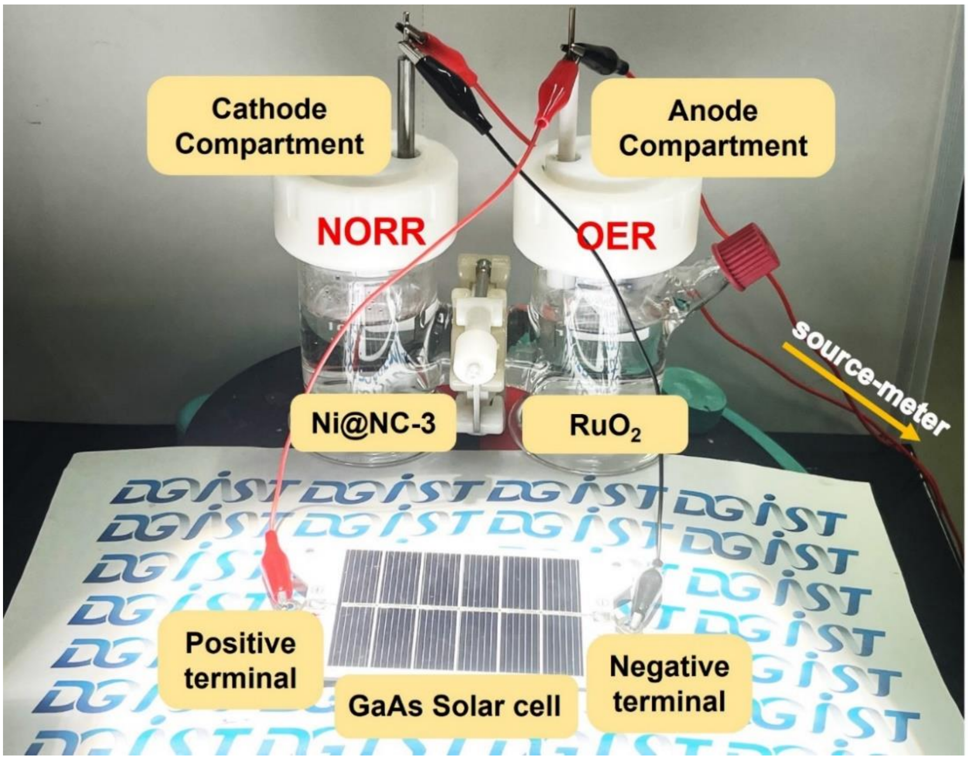

Figure S32. Photograph of the cell setup for the PV-electrolyzer used in NORR-OER electrolysis.

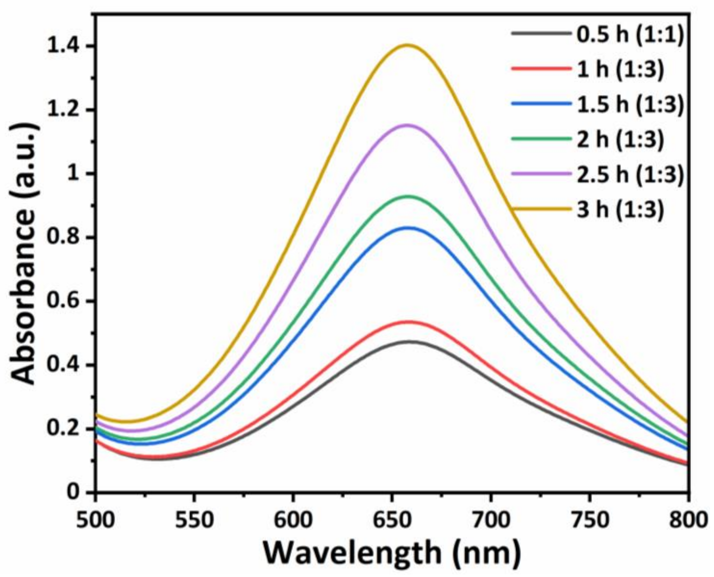

Figure S33. UV-vis absorbance of the electrolyte aliquots stained with indophenol every 0.5 h. (The value in the bracket corresponds to the ratio of catholyte aliquots and bare electrolyte used for dilution)

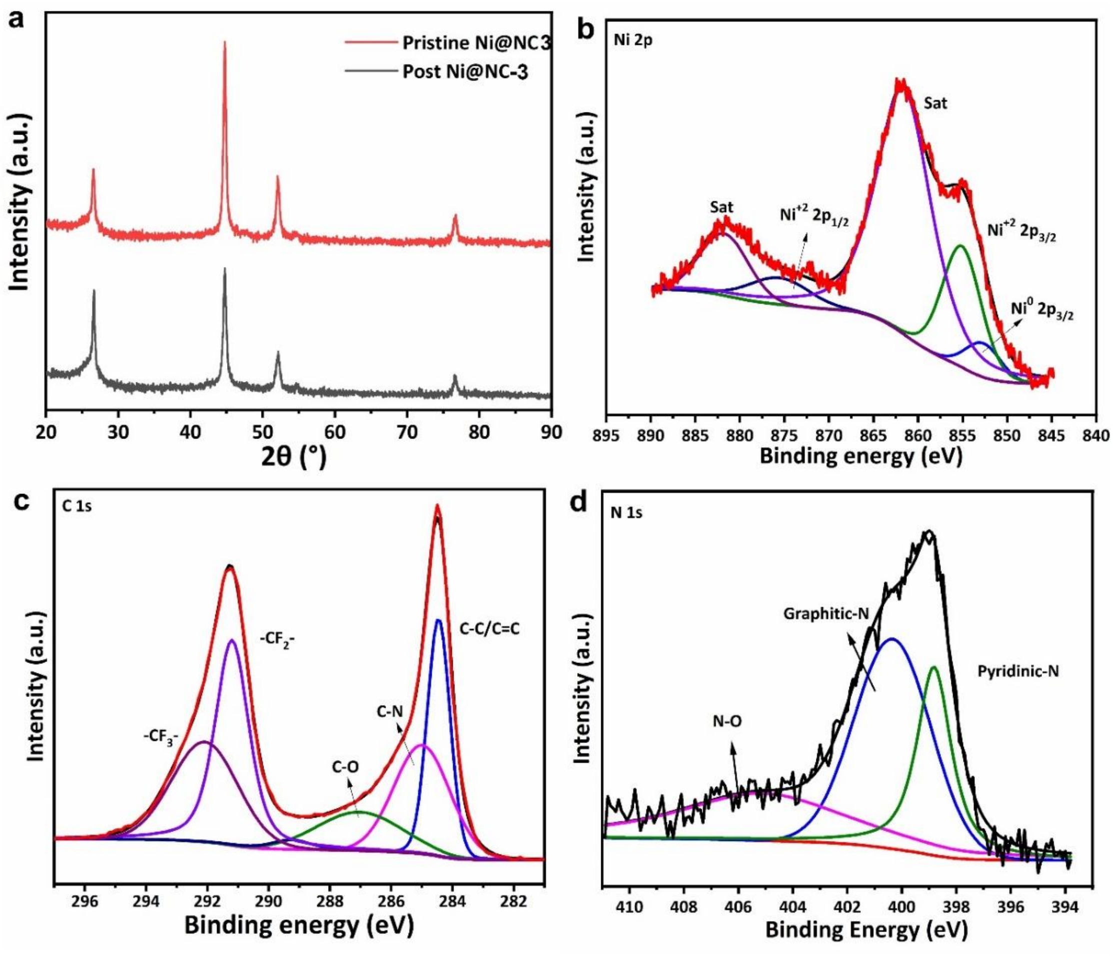

Figure S34. Post characterization of the core-shell Ni@NC-3 catalysts. a) XRD pattern. High- resolution XPS spectra of b) Nickel (Ni 2p), c) Carbon (C1s), and d) Nitrogen (N1s).

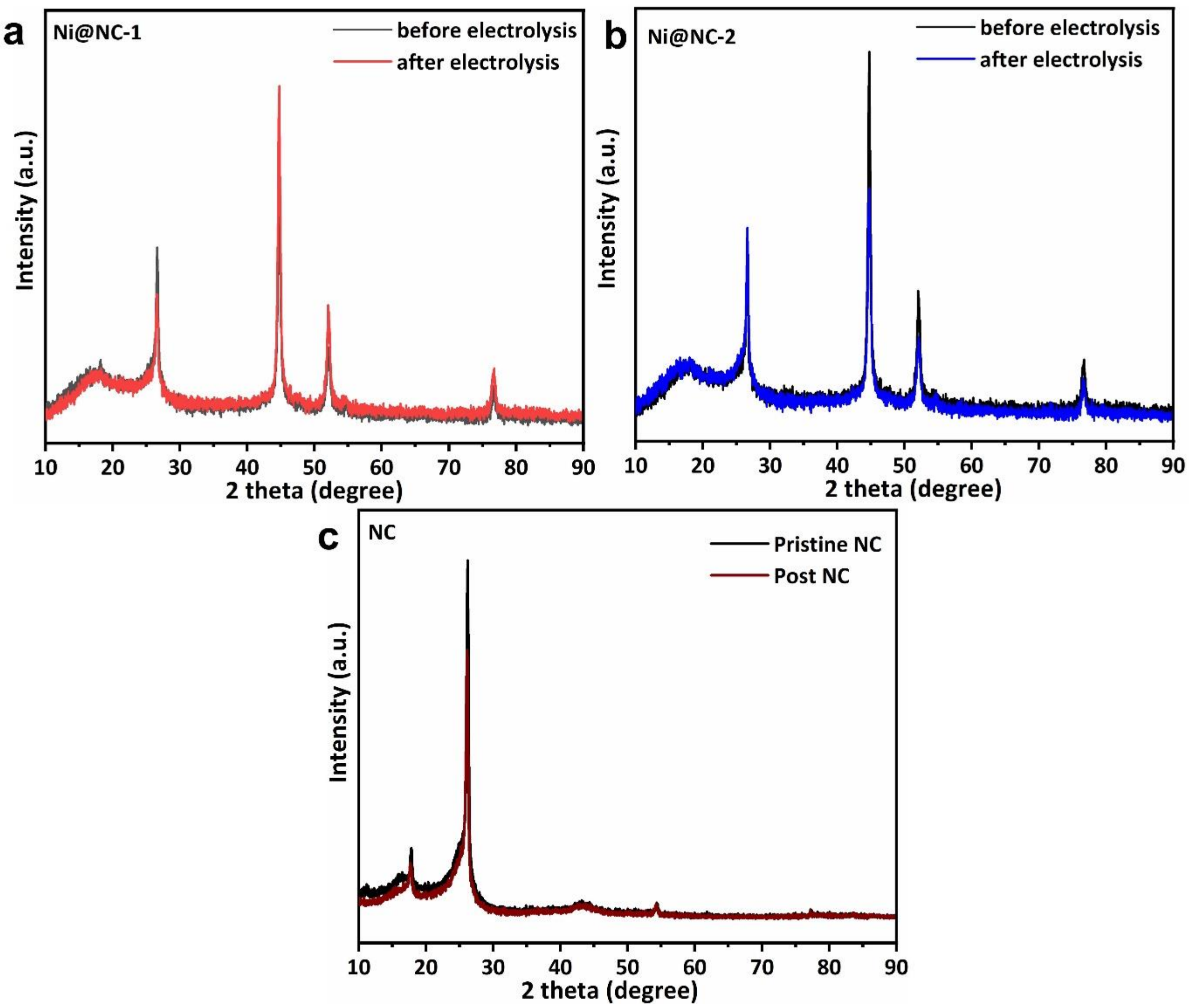

Figure S35. XRD pattern before and after electrolysis of a) Ni@NC-1, b) Ni@NC-2, and c) NC catalysts.

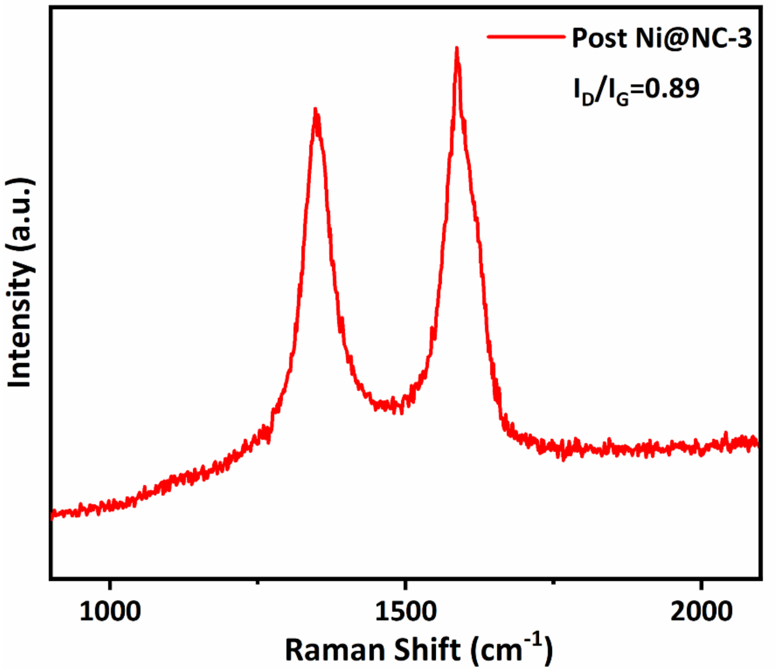

Figure S36. The post-Raman analysis of Ni@NC-3 after stability test.

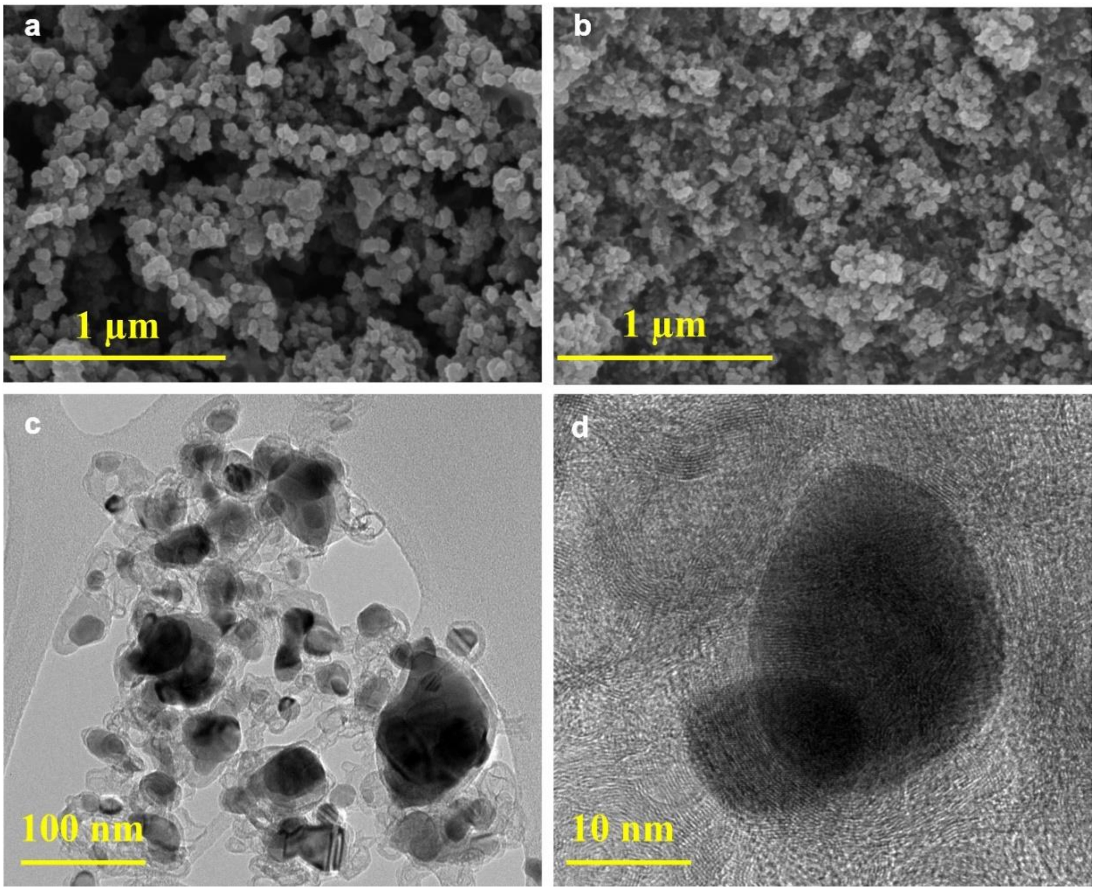

Figure S37. FESEM image of Ni@NC-3 coated on GDE a) before and b) after the stability test, c,d) TEM images of Ni@NC-3 after the stability test

Table S1. Elemental analysis of NiNi-PBA precursor and Ni@NC catalysts.

Catalyst	C [%]	N [%]
NiNi-PBA	17.38	19.82
Ni@NC-1	25.74	3.82
Ni@NC-2	25.82	2.39
Ni@NC-3	26.07	1.64

Table S2. BET surface area and BJH pore size distributions are derived from Figure S13.

Catalyst	Surface area [m^2 g^{-1}]	Pore Size [nm]
Ni@NC-1	71	31.7
Ni@NC-2	86	24.3
Ni@NC-3	90	19.9

Table S3. Calculation of ECSA and Roughness factor.

Catalyst	E_{DL} [μF]	ECSA [cm^2]	Roughness Factor
Ni@NC-1	15	0.42	0.42
Ni@NC-2	15.9	0.45	0.45
Ni@NC-3	18.2	0.52	0.52

Table S4. Nickel quantification in Ni@NC catalysts using ICP-OES

**Catalyst**	**Ni [ppm]**
Ni@NC-1	0.551
Ni@NC-2	0.584
Ni@NC-3	0.594

Table S5. Comparison of electrocatalytic NO performance of Ni@NC with reported aqueous-based NORR electrocatalysts.

S. No.	Catalysts	Electrolyte	NH_3 yield rate	FE_{NH3} [%]	Potential [Vs RHE]	Reference
**1**	**Ni@NC**	**0.1 M HCl**	**34.6 μmol cm^{-2} h^{-1}**	**72.3**	**0.16**	**This work**
2	FeNC	0.1 M $HClO_4$	20.2 μmol cm^{-2} h^{-1}	~5.1	-0.2	[5]
3	Ni_2P/CP	0.1 M HCl	33.5 μmol cm^{-2} h^{-1}	76.9	-0.2	[6]
4	Ag	0.5 M PBS + 50 mM EFeMC	0.28 mol m^{-2} h^{-1}	~100	−0.165	[7]
5	NiO/TM	0.1 M Na_2SO_4 + 0.05 mM Fe^{2+}-EDTA	125.3 μmol cm^{-2} h^{-1}	90	−0.6	[8]
6	Single atom Nb	0.1 M HCl	295.2 μmol cm^{-2} h^{-1}	77.1	-0.6	[9]
7	MoS_2/GF	0.1 M HCl + 0.5 mM Fe(II)SB	99.6 μmol cm^{-2} h^{-1}	~25	−0.7	[10]
8	Cu foam	0.25 M Li_2SO_4	517.1 μmol cm^{-2} h^{-1}	93.5	-0.9	[11]
9	Cu foil	0.25 M Li_2SO_4	95.0 μmol cm^{-2} h^{-1}	61.9		
10	Pt foil	0.25 M Li_2SO_4	99.4 μmol cm^{-2} h^{-1}	24.1		
11	Cu nanoparticle	0.1 M NaOH + 0.9 M $NaClO_4$	1246 μmol cm^{-2} h^{-1}	78	0.1	[12]

References

[1] A. Sivanantham, P. Ganesan, L. Estevez, B. P. McGrail, R. K. Motkuri, S. Shanmugam, Advanced Energy Materials **2018**, 8, 1702838.

[2] P. L. Searle, Analyst **1984**, 109, 549.

[3] G. W. Watt, J. D. Chrisp, Analytical Chemistry **1952**, 24, 2006.

[4] D. S. Frear, R. C. Burrell, Analytical Chemistry **1955**, 27, 1664.

[5] D. H. Kim, S. Ringe, H. Kim, S. Kim, B. Kim, G. Bae, H.-S. Oh, F. Jaouen, W. Kim, H. Kim, C. H. Choi, Nature Communications **2021**, 12, 1856.

[6] T. Mou, J. Liang, Z. Ma, L. Zhang, Y. Lin, T. Li, Q. Liu, Y. Luo, Y. Liu, S. Gao, H. Zhao, A. M. Asiri, D. Ma, X. Sun, Journal of Materials Chemistry A **2021**, 9, 24268.

[7] D. Kim, D. Shin, J. Heo, H. Lim, J.-A. Lim, H. M. Jeong, B.-S. Kim, I. Heo, I. Oh, B. Lee, M. Sharma, H. Lim, H. Kim, Y. Kwon, ACS Energy Letters **2020**, 5, 3647.

[8] P. Liu, J. Liang, J. Wang, L. Zhang, J. Li, L. Yue, Y. Ren, T. Li, Y. Luo, N. Li, B. Tang, Q. Liu, A. M. Asiri, Q. Kong, X. Sun, Chemical Communications **2021**, 57, 13562.

[9] X. Peng, Y. Mi, H. Bao, Y. Liu, D. Qi, Y. Qiu, L. Zhuo, S. Zhao, J. Sun, X. Tang, J. Luo, X. Liu, Nano Energy **2020**, 78, 105321.

[10] L. Zhang, J. Liang, Y. Wang, T. Mou, Y. Lin, L. Yue, T. Li, Q. Liu, Y. Luo, N. Li, B. Tang, Y. Liu, S. Gao, A. A. Alshehri, X. Guo, D. Ma, X. Sun, Angewandte Chemie International Edition **2021**, 60, 25263.

[11] J. Long, S. Chen, Y. Zhang, C. Guo, X. Fu, D. Deng, J. Xiao, Angewandte Chemie International Edition **2020**, 59, 9711.

[12] B. H. Ko, B. Hasa, H. Shin, Y. Zhao, F. Jiao, Journal of the American Chemical Society **2022**, 144, 1258.
